# Comparative analysis of *Diospyros* (Ebenaceae) plastomes: Insights into genomic features, mutational hotspots, and adaptive evolution

**DOI:** 10.1002/ece3.10301

**Published:** 2023-07-12

**Authors:** Yue Huang, Qing Ma, Jing Sun, Li‐Na Zhou, Chan‐Juan Lai, Pan Li, Xin‐Jie Jin, Yong‐Hua Zhang

**Affiliations:** ^1^ College of Life and Environmental Science Wenzhou University Wenzhou China; ^2^ College of Biology and Environmental Engineering Zhejiang Shuren University Hangzhou China; ^3^ Laboratory of Systematic & Evolutionary Botany and Biodiversity, College of Life Sciences Zhejiang University Hangzhou China

**Keywords:** *Diospyros*, genetic diversity, hyper‐variable region, plastome

## Abstract

*Diospyros* (Ebenaceae) is a widely distributed genus of trees and shrubs from pantropical to temperate regions, with numerous species valued for their fruits (persimmons), timber, and medicinal values. However, information regarding their plastomes and chloroplast evolution is scarce. The present study performed comparative genomic and evolutionary analyses on plastomes of 45 accepted *Diospyros* species, including three newly sequenced ones. Our study showed a highly conserved genomic structure across the *Diospyros* species, with 135–136 encoding genes, including 89 protein‐coding genes, 1–2 pseudogenes (Ψ*ycf*1 for all, Ψ*rps*19 for a few), 37 tRNA genes and 8 rRNA genes. Comparative analysis of *Diospyros* identified three intergenic regions (ccs*A‐ndhD*, *rps*16‐*psb*K and *pet*A‐*psb*J) and five genes (*rpl*33, *rpl*22, *petL*, *psaC* and *rps*15) as the mutational hotspots in these species. Phylogenomic analysis identified the phylogenetic position of three newly sequenced ones and well supported a monophylogenetic (sub)temperate taxa and four clades in the pantropical taxa. The analysis codon usage identified 30 codons with relative synonymous codon usage (RSCU) values >1 and 29 codons ending with A and U bases. A total of three codons (UUA, GCU, and AGA) with highest RSCU values were identified as the optimal codons. Effective number of codons (ENC)‐plot indicated the significant role of mutational pressure in shaping codon usage, while most protein‐coding genes in *Diospyros* experienced relaxed purifying selection (*d*_N_/*d*_S_ < 1). Additionally, the *psb*H gene showed positive selection (*d*_N_/*d*_S_ > 1) in the (sub)temperate species. Thus, the results provide a meaningful foundation for further elaborating *Diospyros*'s genetic architecture and taxonomy, enriching genetic diversity and conserving genetic resources.

## Graphical abstract

Thus, the results provide a foundation for elaborating Diospyros's genetic architecture and taxonomy, conserving genetic diversity and enriching genetic resources.

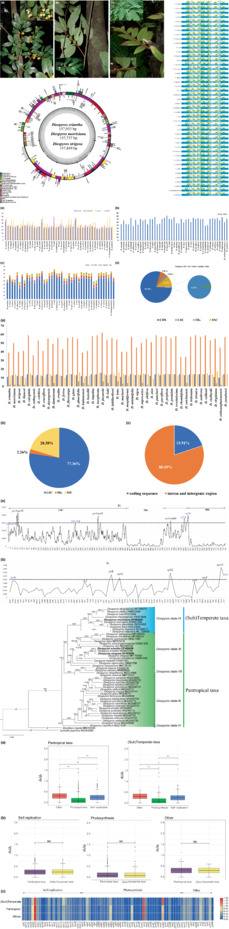

## 1 INTRODUCTION

*Diospyros* (Ebenaceae) is a genus well‐known for hardwood, delicious fruits and medicines (Lee et al., 1996; Lin et al., 2020; Luo et al., 2021; Wallnöfer, 2001; White, 1956). *Diospyros* is the largest genus of the Ebenaceae family, with about 500 evergreen or deciduous shrub and tree species distributed in tropical and temperate regions (Lee et al., 1996; The Plant List, 2002). The genus is characterized by male cymose inflorescence, solitary female flowers, fleshy berries with enlarged persistent calyx at the base, and a dioecious breeding system (Lee et al., 1996). However, the morphological similarities make it difficult to distinguish the species, hindering research and economic development.

Previous infrafamilial classification based on a phylogenetic approach (multilocus) proposed that Ebenaceae consists of two subfamilies, Lissocarpoideae and Ebenoideae, and four genera, *Lissocarpa*, *Euclea*, *Royena*, and *Diospyros* (Duangjai et al., 2006). Previous studies found that *Diospyros* belongs to the Ebenoideae subfamily (Ebenaceae) and is closely associated with *Euclea* Murray and *Royena* L. (Duangjai et al., 2006, 2009; Fu et al., 2016; Li et al., 2018; Linan et al., 2019; Samuel et al., 2019). Within the genus, about 11 (or 12) clades were supported by molecular phylogenetic studies based on multilocus or genomes (Duangjai et al., 2006, 2009; Linan et al., 2019). A previous study has established that the island *Diospyros* species have been shaped by ancestral bottlenecks, rapid and recent radiations in phenotypic characters, and repeated and convergent evolution of potentially adaptive traits during the diversification (Fernández‐Mazuecos et al., 2020). The island *Diospyros* taxa (New Caledonia) also experienced similar evolutionary pressure (Turner et al., 2016). Studies of *Diospyros* about macroevolution of migration among the continents indicated its complex evolutionary history of species diversity (Duangjai et al., 2009; Linan et al., 2019). However, there is little attention paid to adaptive evolution of some *Diospyros* species (clade) shifting along latitude in different climatic zones. Unlike the pantropical clades of *Diospyros*, previous studies showed that the (sub)temperate clade is distributed in higher latitude zones from subtropical to temperate including *D. virginiana* L., *D. kaki* Thunb., *D. lotus* L., and so forth (Duangjai et al., 2009; Linan et al., 2019; Yonemori et al., 2008). In order to adapt to environmental conditions of high‐latitude or high‐elevation, *Diospyros* taxa of (sub)temperate clade are usually deciduous with broad thin‐leathery leaves (Lee et al., 1996; Tang et al., 2014). There are already examples of selective pressures associated with habitats seem to have caused the rapid evolution of genes involved in cold response in *Cardamine* (Ometto et al., 2012), in high‐altitude response in *Dysosma* (Ye et al., 2018) or in sunlight preferences in *Oryza* (Gao et al., 2019). Therefore, on the basis of previous molecular phylogenetic researches, it is of great significance to study the adaptive evolution of *Diospyros* along latitude in different climatic zones by using new molecular markers such as plastomes.

The structurally stable and maternally inherited plastomes with low recombinant levels play a pivotal role in phylogenetic and evolutionary studies (Jansen et al., 2007; Wicke et al., 2011; Xia, Liao, et al., 2022; Xia, Liu, et al., 2022). The genes in plastomes primarily encode proteins related to photosynthesis and other biochemical pathways, including starch storage, nitrogen and sulfate metabolism, and chlorophyll, carotenoid, or fatty acid synthesis (Mohanta et al., 2020; Wicke et al., 2011). Moreover, plastomes are conserved in terms of genomic structures and substitution rates among most Angiosperms, which make plastomes a widely used molecular marker. Additionally, several studies have detected positive selection signals in plastid genes during evolution. For example, accelerated evolutionary rates of *mat*K (Maturase K) in the low‐altitude and recently derived lineages of *Dysosma* are related to the adaptation of the genus to high‐altitude environments (Ye et al., 2018). Furthermore, analysis of the *d*_N_/*d*_S_ ratios of Cardamineae suggested positive selection on the *ycf*2 (hypothetical chloroplast RF21) gene in watercress, possibly allowing the species to adapt to specific living environments (Yan et al., 2019). Most plastid genes are under selection pressure due to their significant roles in maintaining essential cellular functions and, therefore, often retain the adaptive characteristics during evolution (Wicke et al., 2011). Additionally, the codon usage bias in plastomes serves as a suitable strategy for identifying the principal evolutionary driving forces (Gao et al., 2022; Jiang et al., 2014; Kapralov & Filatov, 2007). For example, the effective number of codons (ENC)‐plot showing deviations from the expected curve for a few genes suggested that apart from natural selection, mutational pressure also played a major role in shaping codon usage in *Helianth us annuus* (Gao et al., 2022). These findings have demonstrated that the genetic diversity in plastomes provides useful information about plants' adaptive evolution.

The present study tried to study the adaptive evolution of *Diospyros* using plastomes. We assembled plastomes of 45 accepted *Diospyros* species including three newly sequenced ones with two different climatic zones: (sub)temperate taxa (clade IX in Duangjai et al., 2009), subtemperate to temperate regions of the Northern Hemisphere and pantropical taxa (clade III, IV, VII & XI in Duangjai et al., 2009; New Caledonia and general pantropical taxa from Asia and Africa). The specific objectives of the study were to (1) evaluate the plastome variations in *Diospyros* among the 45 species; (2) develop new plastid DNA ptDNA markers for DNA barcoding; (3) perform the phylogenetic analysis for *Diospyros* species identification using the complete plastomes and (4) analyze the codon usage bias of plastid genes and scan for candidate genes that could evolve under Darwinian selection in different climatic zones.

## 2 MATERIALS AND METHODS

### 2.1 DNA extraction

**FIGURE 1:**
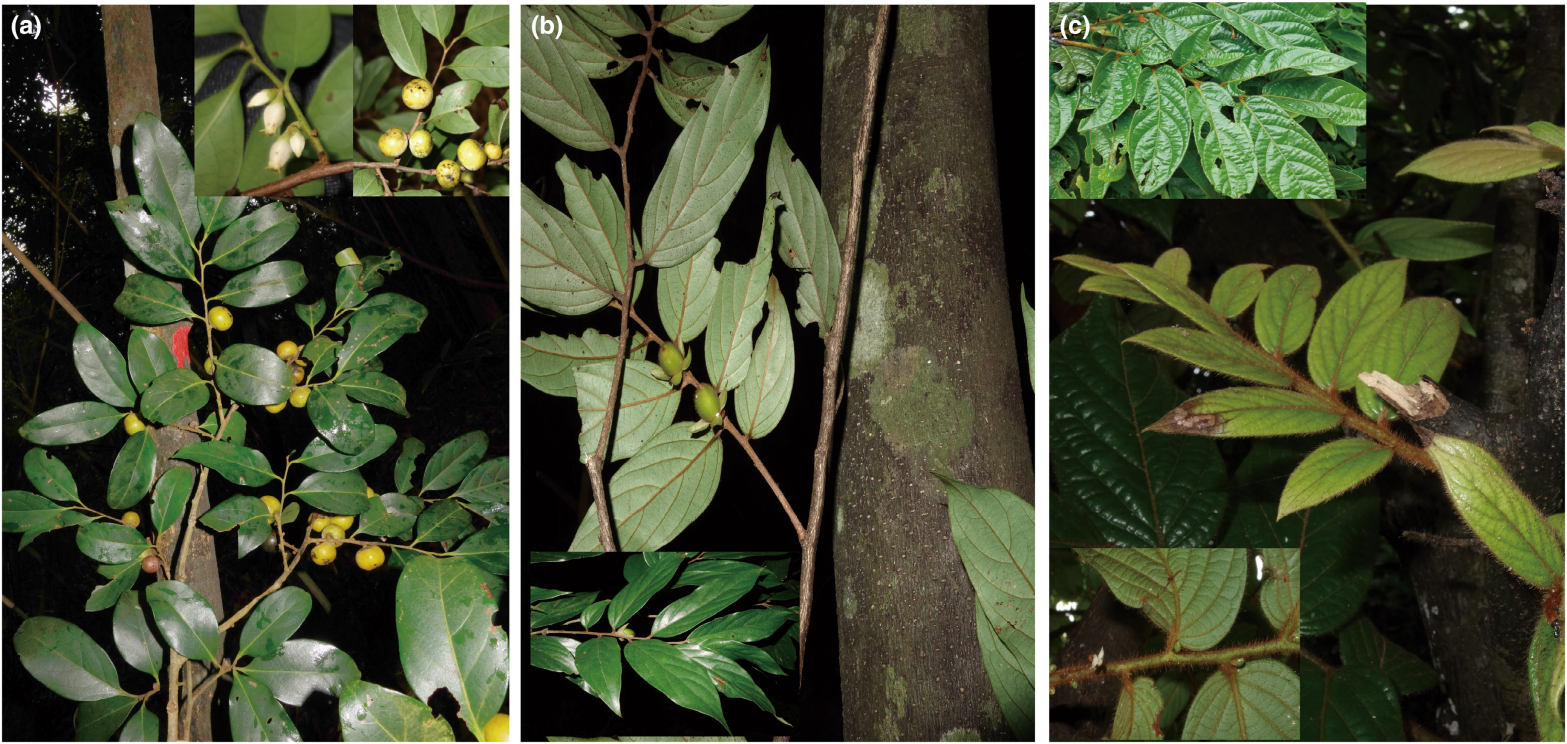
Morphologies of three newly sequenced *Diospyros* species. (a) *Diospyros morrisiana* Hance [Heishiding, Zhaoqing, Guangdong, China, by Y. H. Zhang]; (b) *Diospyros eriantha* Champ. ex Benth. [Heishiding, Zhaoqing, Guangdong, China, by Y. H. Zhang]; (c) *Diospyros strigosa* Hemsl. [Wuzhizhou Island, Hainan, China, by Y. H. Zhang].

The plastomes of three *Diospyros* species (Figure 1), *D. strigosa* Hemsl., *D. morrisiana* Hance, and *D. eriantha* Champ. ex Benth., were newly sequenced in this study collected from South China Botanical Garden and Hainan Province (Table 1). The specimens have been deposited in the Herbarium of Wenzhou University (Table 1). Genomic DNA was extracted from approximately 20 mg of silica‐dried leaves using DNA Plantzol Reagent (Hangzhou Lifefeng Biotechnology Co., Ltd). The quality and quantity of the extracted DNA samples were assessed using agarose gel electrophoresis and ultraviolet‐microspectrophotometry.

**TABLE 1:** Geographic information and specimen voucher number of the *Diospyros* species sequenced in this study.

Species	Voucher no.	Plastome	Locality
*Diospyros eriantha*	ZYH18080302	OP480008	South China National Botanical Garden, Guangzhou, Guangdong, China (27.919° N, 120.694° E)
*Diospyros morrisiana*	ZYH18072102	OP485441	Jianfengling, Ledong, Hainan, China (18.727° N, 108.895° E)
*Diospyros strigosa*	ZYH18080301	OP480009	South China National Botanical Garden, Guangzhou, Guangdong, China (27.919° N, 120.694° E)

### 2.2 Genome sequencing, assembly, and annotation

Approximately 1 μg of the extracted DNA with a concentration higher than 12.5 ng/μL was used for plastome sequencing at the Beijing Genomics Institute (BGI). Before sequencing, total DNA was sheared into fragments shorter than 800 bp. The DNA fragments' quality was evaluated using Agilent Bioanalyzer 2100 (Agilent Technologies), and the pooled library was sequenced on an Illumina HiSeq X10 platform to obtain 150 bp long raw reads.

The raw reads were filtered by removing the sequences with a Phred score lower than 30, and the remaining ones were used for genome assembly using GetOrganelle toolkit (Jin et al., 2020). The command lines used for the assembly were as follows: get_organelle_reads.py ‐1 forward.fq ‐2 reverse.fq ‐o plastome_output ‐R 15 ‐k 21,45,65,85,105 ‐F plant_cp. The newly sequenced plastomes of *Diospyros* species were annotated with Geneious Prime 2021 (Biomatters), using the plastome sequence of *D. virginiana* L. (GenBank accession no. MF288577) as the reference. The CPGAVAS2 web server (http://www.herbalgenomics.org/cpgavas) predicted the types and structures of all the protein‐coding and noncoding genes in the plastome. The location of the start and stop codons, exon‐intron boundaries, and the tRNA gene length and types were confirmed by comparing the annotation results from CPGAVAS2 and Geneious Prime 2021. Finally, the plastome maps for the newly sequenced species were drawn using the online tool OrganellarGenomeDRAW (Lohse et al., 2007). Plastomes of 42 other *Diospyros* species and two outgroups (*Manilkara zapota*: MN295595 and *Camellia japonica*: MG543990; Table 2, Figure 7) were downloaded from NCBI GenBank repository and re‐annotated using the earlier method. According to the climatic zones of *Diospyros* species, it can be divided into (sub)temperate taxa (eight species) and pantropical taxa (37 species; Table 2).

**TABLE 2:** The features of plastomes of 45 *Diospyros* species and 2 outgroup species.

Species	GenBank	Climatic zone	Total (bp)	LSC (bp)	SSC (bp)	IR (bp)	CDS (bp)	Gene	CDS	Pseudo	tRNA	rRNA
***D. eriantha***	OP480008	Pantropical	157,933	87,181	18,474	26,139	80,520	136	89	2	37	8
***D. morrisiana***	OP485441	(Sub)Temperate	157,737	87,106	18,451	26,090	80,259	136	89	2	37	8
***D. strigosa***	OP480009	Pantropical	157,849	87,158	18,467	26,112	80,481	136	89	2	37	8
*D. blancoi*	KX426216	Pantropical	157,745	87,246	18,323	26,088	80,409	136	89	2	37	8
*D. calciphila*	MG049695	Pantropical	157,387	86,878	18,409	26,050	79,800	136	89	2	37	8
*D. cathayensis*	MF288576	Pantropical	157,689	87,176	18,349	26,082	80,475	136	89	2	37	8
*D. celebica*	MN885893	Pantropical	157,643	87,136	18,333	26,087	80,385	136	89	2	37	8
*D. crassiflora*	MZ274087	Pantropical	157,874	87,221	18,475	26,089	80,445	136	89	2	37	8
*D. deyangenesis*	MF288575	(Sub)Temperate	157,934	87,237	18,485	26,106	80,136	136	89	2	37	8
*D. dumetorum*	ON881445	Pantropical	157,788	87,135	18,391	26,131	80,454	136	89	2	37	8
*D. erudita*	MG049697	Pantropical	157,402	86,901	18,381	26,060	79,809	136	89	2	37	8
*D. ferrea*	MG049698	Pantropical	157,398	87,008	18,264	26,063	80,370	136	89	2	37	8
*D. flavocarpa*	MG049699	Pantropical	157,420	86,880	18,420	26,060	79,806	136	89	2	37	8
*D. glans*	MG049701	Pantropical	157,430	86,892	18,418	26,060	79,809	136	89	2	37	8
*D. glaucifolia*	KM504956	(Sub)Temperate	157,593	86,974	18,413	26,103	80,457	135	89	1	37	8
*D. hainanensis*	MH778100	Pantropical	157,999	87,523	18,322	26,077	80,391	136	89	2	37	8
*D. hasseltii*	ON881551	Pantropical	157,857	87,232	18,411	26,107	80,436	136	89	2	37	8
*D. impolita*	MG049702	Pantropical	157,416	86,898	18,414	26,052	79,803	136	89	2	37	8
*D. inexplorata*	MG049703	Pantropical	157,384	86,873	18,411	26,050	79,800	136	89	2	37	8
*D. jinzaoshi*	KM522848	(Sub)Temperate	157,321	86,919	18,174	26,114	80,451	136	89	2	37	8
*D. kaki*	KT223565	(Sub)Temperate	157,784	87,099	18,533	26,076	80,259	135	89	1	37	8
*D. labillardierei*	MG049704	Pantropical	157,422	86,911	18,403	26,054	79,797	136	89	2	37	8
*D. lotus*	KM522849	(Sub)Temperate	157,590	86,944	18,416	26,115	80,457	136	89	2	37	8
*D. maclurei*	MH778101	Pantropical	157,946	87,387	18,397	26,081	78,348	136	89	2	37	8
*D. mespiliformis*	MZ274088	Pantropical	157,246	86,794	18,308	26,072	80,346	136	89	2	37	8
*D. minimifolia*	MG049707	Pantropical	157,450	86,927	18,403	26,060	79,809	136	89	2	37	8
*D. nigra*	MZ929416	Pantropical	157,186	86,610	18,386	26,095	80,433	136	89	2	37	8
*D. nigrocortex*	ON881766	Pantropical	157,441	86,850	18,443	26,074	79,956	136	89	2	37	8
*D. oleifera*	KM522850	(Sub)Temperate	157,724	87,056	18,522	26,073	80,253	135	89	1	37	8
*D. olen*	MG049708	Pantropical	157,589	86,970	19,127	25,746	80,460	136	89	2	37	8
*D. pancheri*	MG049709	Pantropical	157,424	86,892	18,410	26,061	79,800	136	89	2	37	8
*D. parviflora*	MG049713	Pantropical	157,467	86,910	18,437	26,060	77,859	136	89	2	37	8
*D. perplexa*	MG049717	Pantropical	157,405	86,874	18,411	26,060	79,800	136	89	2	37	8
*D. pustulata*	MG049719	Pantropical	157,506	86,949	18,437	26,060	79,809	136	89	2	37	8
*D. revolutissima*	MG049721	Pantropical	157,444	86,906	18,418	26,060	79,809	136	89	2	37	8
*D. rhombifolia*	MF288578	Pantropical	157,368	87,223	18,325	25,910	80,496	136	89	2	37	8
*D. sutchuensis*	OK641590	Pantropical	157,917	87,303	18,392	26,111	80,433	136	89	2	37	8
*D. tridentata*	MG049723	Pantropical	157,479	86,941	18,418	26,060	79,794	136	89	2	37	8
*D. trisulca*	MG049724	Pantropical	157,388	86,870	18,414	26,052	79,800	136	89	2	37	8
*D. umbrosa*	MG049726	Pantropical	157,447	86,917	18,420	26,055	79,815	136	89	2	37	8
*D. veillonii*	MG049727	Pantropical	157,399	86,876	18,419	26,052	79,809	136	89	2	37	8
*D. vieillardii*	MG049728	Pantropical	157,544	86,999	18,409	26,068	80,329	136	89	2	37	8
*D. virginiana*	MF288577	(Sub)Temperate	157,761	87,089	18,444	26,114	80,385	136	89	2	37	8
*D. xishuangbannaensis*	ON881559	Pantropical	157,379	87,084	18,339	25,978	80,346	135	89	1	37	8
*D. yaouhensis*	MG049731	Pantropical	157,409	86,874	18,415	26,060	79,803	136	89	2	37	8
*Camellia japonica*	MG543990	Outgroup	157,047	86,656	18,281	26,055	79,857	136	89	2	37	
*Manilkara zapota*	MN295595	Outgroup	158,386	87,745	18,443	26,099	80,472	136	89	2	37	

*Note*: The newly sequenced data is shown in bold.

### 2.3 Plastome comparison

The GenBank accession numbers of the plastomes of the 45 *Diospyros* species used for comparative analyses are shown in Table 2. The plastome sequences of these 45 *Diospyros* species were aligned using the LAGAN model implemented in the mVISTA software to evaluate the degree of variation (Frazer et al., 2004), using default parameters and *D. eriantha* as the reference. The rearrangement in the sequences was detected using the whole genome alignment tool Mauve implemented in Geneious Prime 2021 (Darling et al., 2004).

### 2.4 Detection of repeated sequences

Repeated sequences are essential components of the gene regulatory network; they are identical or complementary nucleotide fragments distributed throughout the genome. Two large families of repeated sequences, the dispersed repeated sequence (DRS, including forward, reverse, complement, and palindromic sequences) and the tandem repeated sequences (TRS, known as satellite DNA), can be readily recognized based on their distribution pattern in the genome (Sperling & Li, 2013). The satellite DNA refers to the repetitions of short sequences of the DNA and is of three types: macrosatellites, minisatellites, and microsatellites (simple sequence repeats or SSRs; Hoy, 2013). The DRS in the plastomes of 45 *Diospyros* species were predicted with REPuter (Kurtz et al., 2001), and the forward, reverse, palindromic, and complementary repeat sequences were identified using the following parameters: length of repeat unit ≥30 bp, sequence consistency ≥90% (Hamming distance = 3). Meanwhile, the tandem repeats finder (TRF) web server (https://tandem.bu.edu/trf/trf.html) was used to search for TRS in the plastomes using default settings (Benson, 1999), and the MISA software to identify SSRs (Beier et al., 2017), with the minimum length of SSR fragment set to 10 bp and the minimum repetition threshold values for mono‐, di‐, tri‐, tetra‐, penta‐, and hexanucleotide set to 10, 5, 4, 3, 3, and 3, respectively. Finally, all the detected repeat sequences were manually checked and corrected to remove the redundant ones.

### 2.5 Analysis of codon usage

Codon usage bias refers to the unequal usage of synonymous codons in genetic material (Guo et al., 2017; Hershberg & Petrov, 2008; Plotkin & Kudla, 2011). For codon usage analysis, protein‐coding sequences longer than 300 bp with ATG as the start codon were isolated from each plastome. CodonW (http://codonw.sourceforge.net) analyzed the number and types of codons encoding the proteins and calculated the effective number of codons (ENC), the relative synonymous codon usage (RSCU) and the GC3 (guanine and cytosine content at the third codon position) values. Further, the effect of base composition on codon usage bias was evaluated by ENC plotting, with ENC and GC3 values along the *y*‐axis and *x*‐axis. The observed ENC value was compared with the expected ENC value using the following equation (Wright, 1990):

$$\mathrm{ENC}=2+\mathrm{GC}3s+29/\left[\mathrm{GC}3s^{2}+{\left(1-\mathrm{GC}3s\right)}^{2}\right].$$

The effects of gene mutation and natural selection on codon usage bias were evaluated by PR2 plotting with [A3/(A3 + T3)] and [G3/(G3 + C3)] along the *y*‐axis and *x*‐axis: this plot reflects the potential biased usage of A/T and G/C in the third codon position.

### 2.6 Analysis of genetic diversity and selective pressure

The plastomes were aligned using the MUSCLE alignment software implemented in Geneious to screen for the highly divergent regions among the 45 *Diospyros* species (Edgar, 2004). The protein‐coding genes, noncoding genes, and the intergenic regions were extracted from the plastomes to analyze the nucleotide diversity (Pi) among the *Diospyros* species using DnaSP (v5.0; Librado & Rozas, 2009) based on the number of overall mutation and the average nucleotide variation. Then, to evaluate the effect of environmental pressure on the evolution of *Diospyros* species, the nonsynonymous (amino acid–altering) to synonymous (silent) substitution rate ratio (*ω* = *d*_N_/*d*_S_) of all the annotated protein‐coding gene sequences in the plastomes across the phylogeny were calculated, with *ω* = 1, <1 (especially <0.5), and >1, indicating neutral evolution, purifying selection, and positive selection, respectively (Kimura, 1983; Yang & Nielsen, 2002). The branch‐site model (Yang & Nielsen, 2002) was identified positively selected loci from genes in the foreground branch using sequences of *M. zapota* (Sapotaceae).

Furthermore, to examine the selective pressure on the whole plastid genes with different functions, the CDS genes were classified into photosynthesis‐related, self‐replication‐related, and other functional genes (Table S1). To examine if the *d*_N_/*d*_S_ values of CDS genes according to different functional classifications or taxa were significantly different, one‐way analysis of variance (ANOVA) or Mann–Whitney *U* test was performed based on Shapiro–Wilk normality test and Levene test with least significant differences at *p* = .05. Finally, boxplot graphs of the *d*_N_/*d*_S_ values of CDS genes were generated according to different functional classifications or taxa and labeled the significance of the difference between the groups. All analyses were conducted in R version 4.3.0 (https://www.R‐project.org/).

### 2.7 Phylogenomic inference

Phylogenetic analyses were conducted using the complete chloroplast genome sequences of 45 *Diospyros* species, excluding one copy of the inverted repeat, with *M. zapota* (Sapotaceae, a sister to Ebenaceae) and *Camellia japonica* (Theaceae) as outgroups (Duangjai et al., 2009), to explore the evolutionary relationship among the species. Maximum likelihood (ML) and Bayesian inference (BI) methods were employed for the phylogenomic reconstruction of *Diospyros*. The best‐fit nucleotide substitution model for ML and BI analyses was determined by ModelTest (v3.7; Drummond et al., 2002), and the GTR + I + G model was finally selected for phylogenomic analysis. ML and BI analyses were performed using the RAxML‐HPC (v8.1.11; Stamatakis, 2014) and MrBayes (v3.2.3; Ronquist et al., 2013) online tools available from the CIPRES Science Gateway. The ML analysis was conducted with 1000 bootstrap replicates using default settings. For BI analysis, four parallel Markov chains were run simultaneously to iterate 5,000,000 generations, with the first 25% of samples discarded as burn‐in. The phylogenetic trees were sampled every 1000 generations to construct the final consensus tree.

## 3 RESULTS

### 3.1 Genome structure and nucleotide variation

**FIGURE 2:**
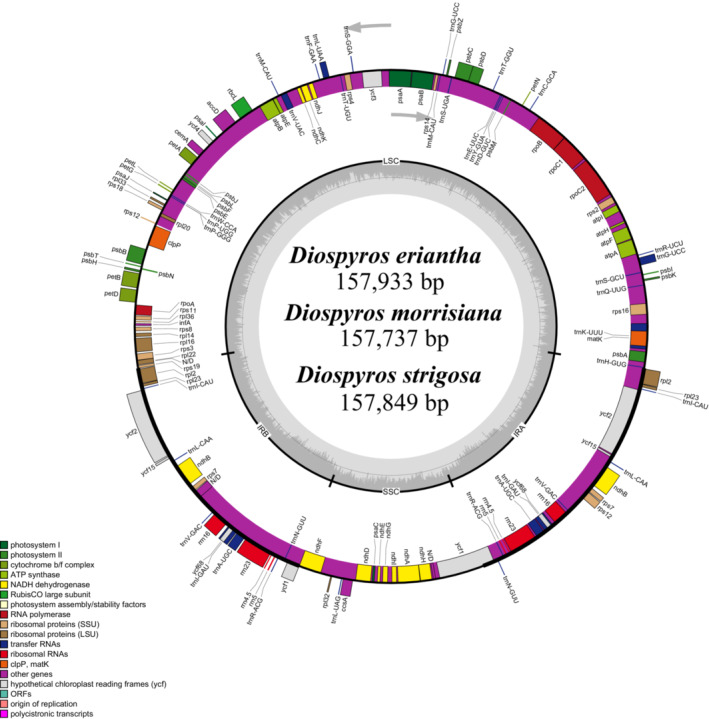
Gene map of *Diospyros* plastomes. Genes inside the outer circle are transcribed clockwise and genes outside the outer circle are transcribed counter‐clockwise. Genes belonging to different functional groups are marked with different color. The dark gray color in the inner circle indicates GC content and the light gray color indicates AT content.

The three newly generated *Diospyros* plastome sequences have been deposited in the GenBank (OP480008, OP480009, OP485441; Table 1). Similar to most angiosperm, these three *Diospyros* species have plastomes with a classic tetrad structure, with two inverted repeats (IR) separated by a large single copy (LSC) region and a small single copy (SSC) region (Figure 2). The plastome sequences of the *Diospyros* species ranged from 157,186 to 157,999 bp, including IRs ranging from 25,746 to 26,139 bp, SSC from 18,174 to 19,127 bp, and LSC from 96,610 to 87,523 bp (Table 2). A total of 135–136 genes, including 89 protein‐coding genes, 1–2 pseudogenes, 37 tRNA genes, and 8 rRNA genes were identified in these species, among which 10 protein‐coding genes, 7 tRNA genes, and 4 rRNA genes were repeated in the two IRs (Table S1). The *ycf*1 in the IRb of all *Diospyros* species (a short Ψ*ycf*1) and the *rps*19 in the IRa region in most *Diospyros* species (a short Ψ*rps*19) were identified as pseudogenes (Table S1). Six tRNAs and nine kinds of protein‐coding genes had one intron, while the *clp*P, *ycf*3, and *rps*12 genes had two (Table S1). The *mat*K gene was found embedded in the intronic region of *trn*K‐UUU, consistent with various other plant taxa. Meanwhile, the trans‐spliced *rps*12 gene, with the 5′ and 3′ ends located in the LSC and IR, had two independent transcription units.

The overall GC content of *Diospyros* species was 37.3%–37.4%, while that of the coding sequences (CDS) was 37.7%–38.2% (Table S2). For all the species, the GC content of IR (43.0%–43.1%) was higher than those of the LSC (35.3%–35.5%) and SSC (30.7%–31.2%) regions (Table S2).

Multiple plastome comparisons among the *Diospyros* species using mVISTA and Mauve alignment showed a high degree of collinearity. The gene organization and distribution patterns in the plastome were highly consistent among the *Diospyros* species (Figure S1). No rearrangement of DNA fragments, including inversion or translocation, was detected among *Diospyros* plastomes sequences (Figure S1). However, slight differences were observed in different regions throughout the plastome sequence. The sequence similarity among *Diospyros* plastomes sequences was much higher in the two IRs, especially the rRNA coding regions. By contrast, the nucleotide mutation rate was high in the noncoding regions, especially the intergenic spacer (IGS) regions (Figures S1 and S2).

**FIGURE 3:**
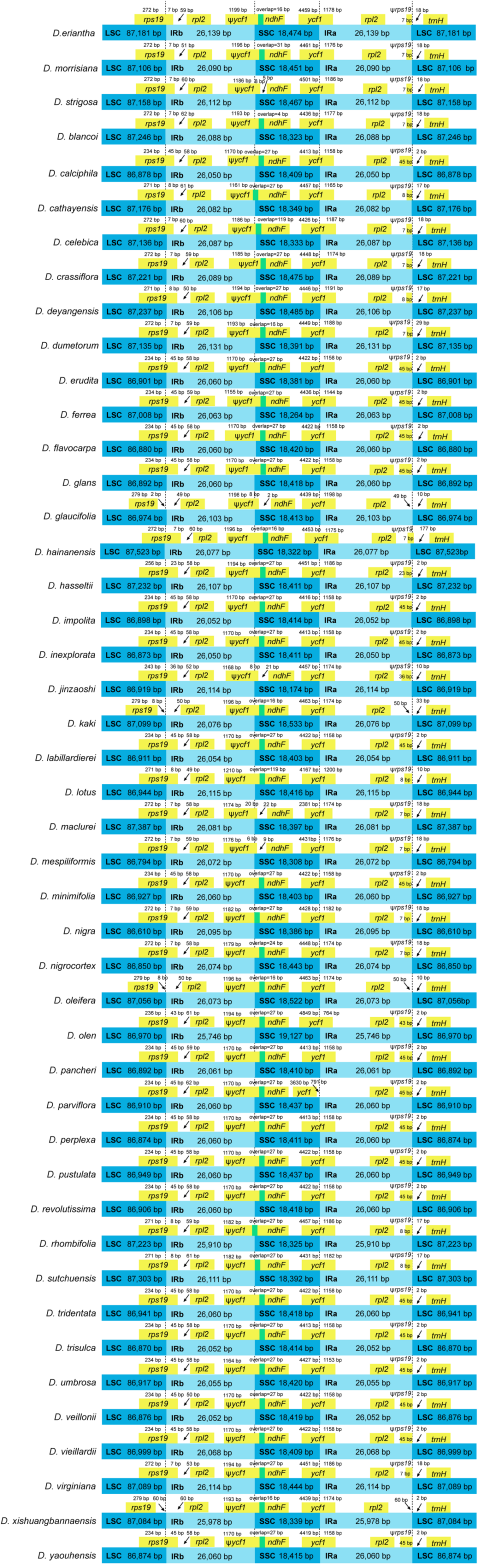
A comparison of IR/SSC and IR/LSC boundaries among 45 *Diospyros* plastomes. Genes close to or spanned the boundaries were shown in yellow boxes. Genes overlap each other were shown in green. IR, inverted repeat; LSC, large single copy; SSC, small single copy.

Contraction and expansion of IR indicate plastome evolution and are correlated with plastome size. The present study found conserved plastome structure in terms of the length of IRs and gene location at the IR/SSC/LSC boundaries among the 45 *Diospyros* species (Figure 3). In all the species, the *rpl*2 and *trn*H genes were located on different sides of the IRa/LSC boundary. The *ycf*1 gene spanned the SSC/IRa boundary with a part of the gene extended to the IRa, forming a pseudogene (Ψ*ycf*1) at the corresponding position near the IRb/SSC boundary. Extension of the short Ψ*ycf*1 fragment into the SSC region was observed in all *Diospyros* species, and an extension of a short portion of *ndh*F into the IRb was observed in *D. cathayensis* and *D. rhombifolia*. The analysis also detected Ψ*ycf*1 and *ndh*F overlap in all species except for *D. mespiliformis*, *D. maclurei*, *D. glaucifolia*, *D. strigosa*, and *D. zhejiangensis*. The *rps*19 gene spanned the LSC/IRb region in all the species, except for *D. glaucifolia*, *D. kaki*, *D. oleifera*, and *D. xishuangbannaensis*, in which the gene was found 2, 8, 8, and 60 bp away from the LSC/IRb junction. In addition, *rps*19 formed a pseudogene (Ψ*rps*19) in all the species except for *D. glaucifolia*, *D. kaki*, *D. oleifera*, and *D. xishuangbannaensis*, where the gene was at the IRa/LSC boundary (Figure 3).

### 3.2 Repetitive sequences in plastomes

**FIGURE 4:**
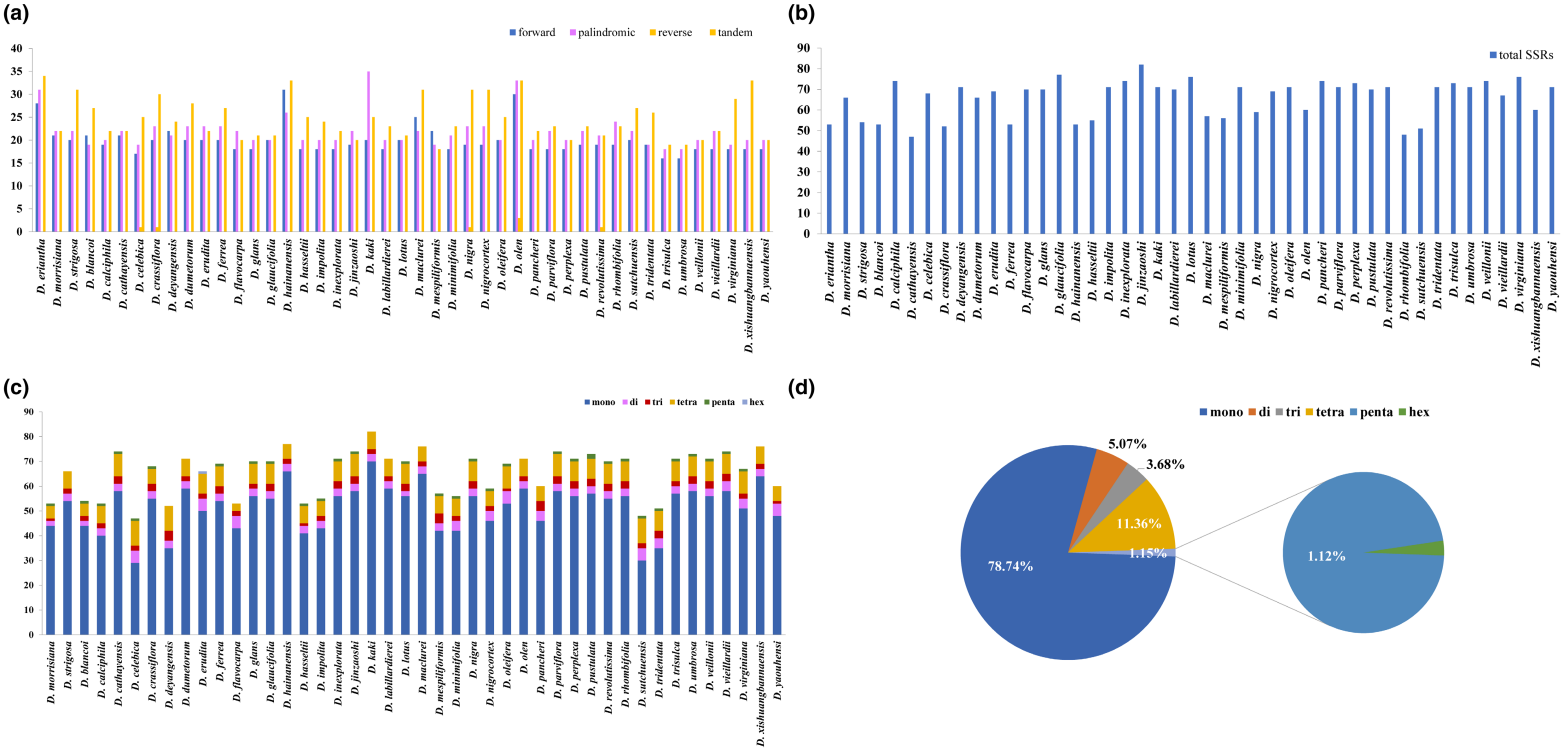
The number and types of repeat sequences in 45 *Diospyros* plastomes. (a) The number of forward, palindromic, and tandem repeats in the plastome of each species. (b) Total number of simple sequence repeats (SSR) in the plastome of each species. (c) The number of different types of SSR in the plastome of each species. (d) The overall proportion of different types of SSR in all the 45 plastomes.

REPuter identified 2988 repeated sequences, including 16–31 forward repeats, 18–35 palindromic repeats, 0–3 reverse repeats, and 18–34 tandem repeats, in the 45 *Diospyros* species (Tables S3 and S4, Figure 4). Among the species, *D. olen* had the maximum (99) forward, palindromic, reverse, and tandem repeats. Tandem repeats were more prevalent and accounted for 37.08% of all the repeat types. On the contrary, reverse repeats were relatively rare and accounted for only 0.23% of the repeat types (Table S4). The length of the dispersed repeats, including forward and palindromic repeats, varied from 30 to 90 bp, while more than half of the tandem repeats were 18–30 bp long (Table S3). The longest tandem repeats were detected in *D. hainanensis* (93 bp) and *D. xishuangbannaensis* (77 bp) and were located in the IGS of *trn*G‐UCC and *trn*R‐UCU (Table S3).

**FIGURE 5:**
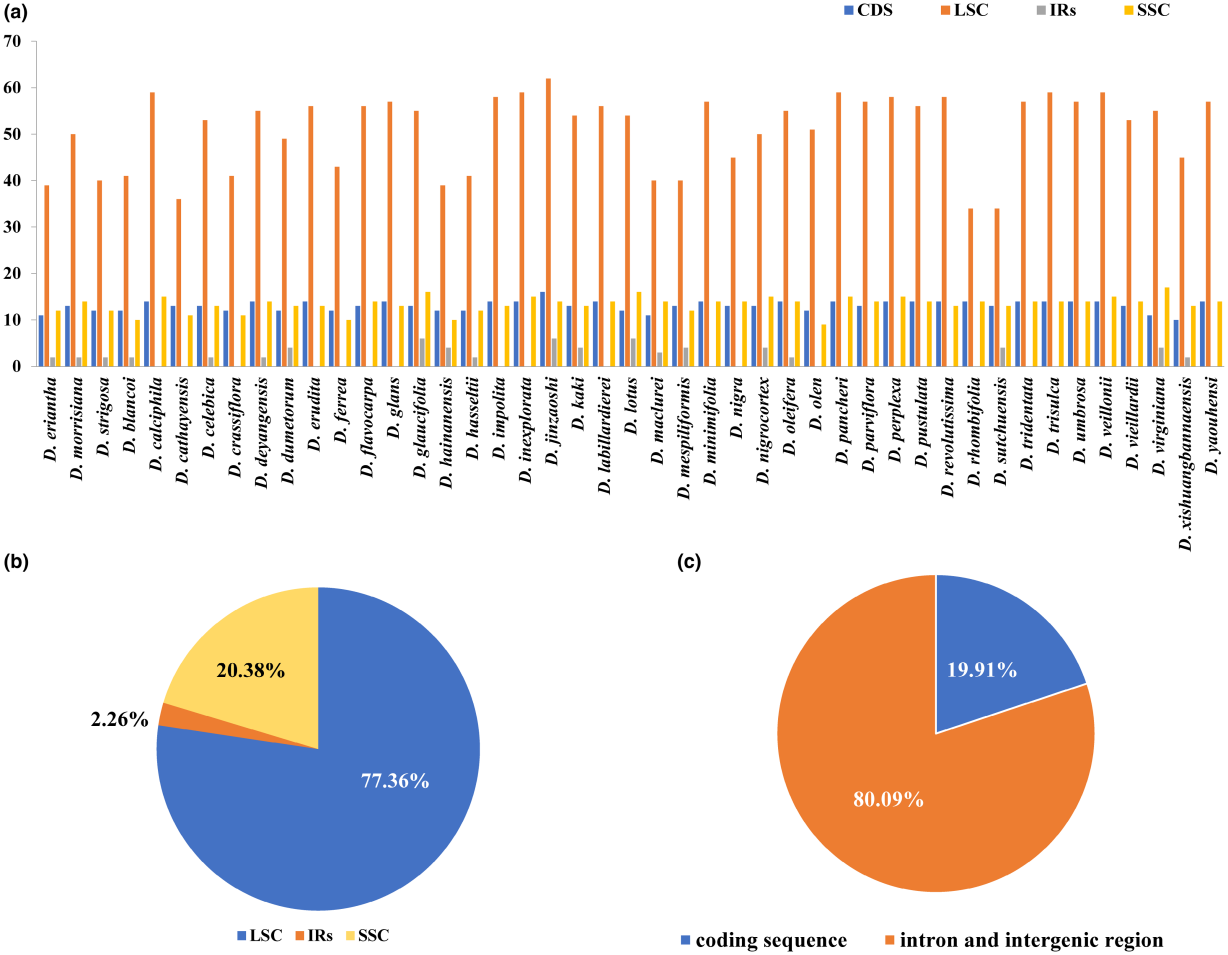
The distribution of simple sequence repeats in 45 *Diospyros* plastomes. (a) The number of SSRs in the large single copy, small single copy, inverted repeat regions and in all the coding sequence (CDS) of the plastome of each species. (b) The overall proportion of SSRs detected in different regions of all the 45 plastomes. (c) The overall proportion of SSRs detected in CDS and non‐coding sequences of all the 45 plastomes.

Additionally, 2959 SSR loci were detected from the 45 *Diospyros* plastomes. The number of SSR loci in each species varied from 47 (*D. cathayensis*) to 82 (*D. zhejiangensis*; Table S4, Figure 4). Most identified SSRs were mononucleotide repeats (78.74%), followed by tetra‐ (11.36%), di‐ (5.07%), trinucleotide (3.68%), and hex‐ (0.03%) repeats (Table S4, Figure 4). Only one hexanucleotide repeat was detected in *D. dumetorum*. Most SSRs (77.36%) were found in the LSC region of the plastome, and only 20.38% and 2.26% were found in the SSC and IR regions, respectively (Tables S3 and S4, Figure 5). In addition, 19.91% of the SSRs were found in the CDS, while the other 80.09% were found in the introns and IGS (Tables S3 and S4, Figure 5).

### 3.3 Nucleotide diversity of plastomes

**FIGURE 6:**
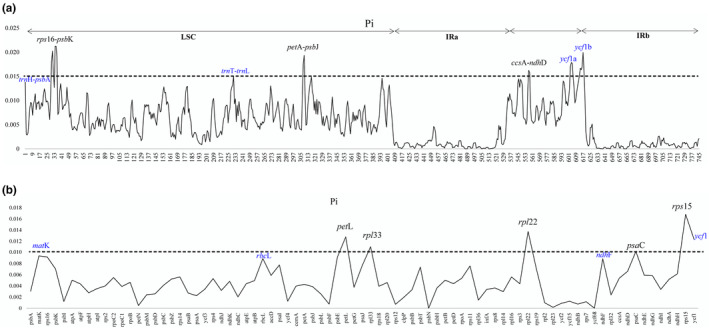
(a) Analysis of the overall sliding window of 45 *Diospyros* plastomes. Sliding window analysis parameters, window length: 600 bp, step size: 200 bp. (b) Analysis of nucleotide polymorphism of protein coding gene. The dash lines indicate Pi = 0.015 in (a) and Pi = 0.010 in (b); intergenic spacers (IGS) and genes in blue font were identified in the previous study (Li et al., 2018).

The alignment of the plastomes discovered six hypervariable regions with a Pi higher than 0.015 (*rps*16‐*psb*K, *trn*T‐*trn*L, *pet*A‐*psb*J, ccs*A‐*ndh*D*, *ycf*1a, *ycf*1b) among the 45 *Diospyros* species (Table S5, Figure 6a). Analysis of the CDS and their nucleotide polymorphisms among the plastomes of the 45 species identified *pet*L, *rpl*33, *rpl*22, *psa*C, *rps*15, and *ycf*1 as the genes with the highest nucleotide polymorphism (Pi > 0.010, Figure 6b). Meanwhile, most nucleotide mutations were detected in the LSC and SSC regions. The nucleotide diversity values (Pi) of the LSC and SSC regions were 0–0.02, while that of the IR was 0–0.01 (Table S5, Figure 6).

Further analysis revealed high variability in the gene spacer, with a Pi value significantly higher than that of the gene‐coding region (CDS; Figure 6). These findings suggest that hypervariable DNA fragments between the different *Diospyros* species could be used as ptDNA barcodes for taxonomic classification, species discrimination, and phylogenetic reconstruction and inference.

### 3.4 Phylogenetic inference

**FIGURE 7:**
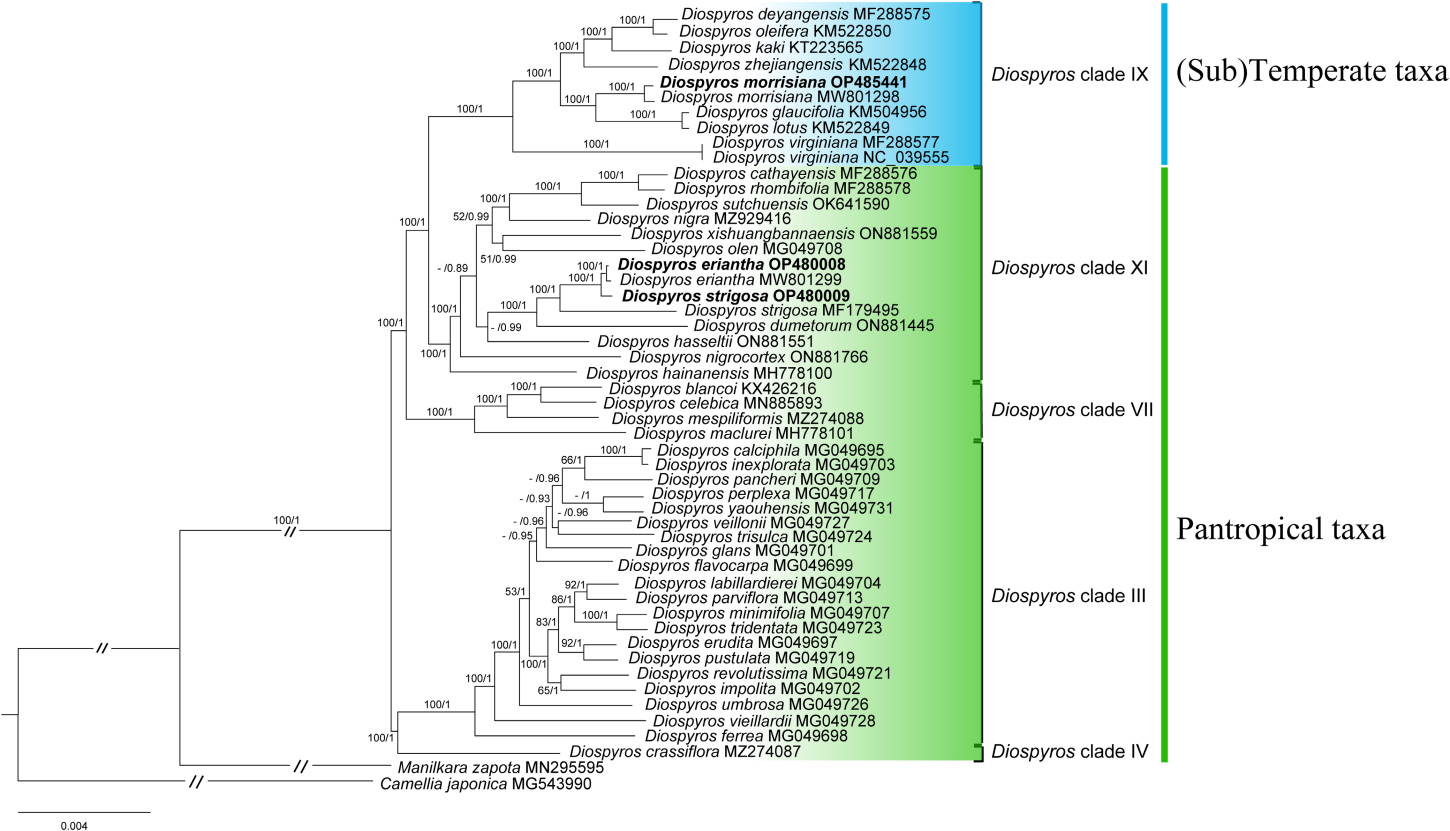
Phylogenetic reconstruction of 45 *Diospyros* species based on complete plastomes using maximum likelihood (ML) and Bayesian inference (BI) analyses. Numbers above each branch represent the bootstrap values (%) from ML analyses, and the posterior probabilities from BI analyses, respectively; ‘–’ indicates support values of <50% or <0.50. Attributions of clade and taxa for *Diospyros* species were referred to Duangjai et al. (2009).

Using Sapotaceae and Theaceae as the outgroups, ML and BI trees of *Diospyros* species based on the complete plastome (excluding one copy of the inverted repeat) showed nearly identical topologies (Figure 7). Both trees revealed that *D. morrisiana* was clustered with *D. glaucifolia* and *D. lotus* (Figure 7). Meanwhile, *D. kaki*, *D. oleifera*, and the two cultivated species *D. deyangensis* and *D. zhejiangensis* (named *‘*jinzaoshi’; Tang et al., 2019) formed the other subclade. Then, *D. virginiana* combining all above species formed a clade, which all of them are natively distributed in temperate and subtemperate zone of north hemisphere. In the pantropical taxa, there was a close relationship between newly sequenced species, *D. eriantha* and *D. strigosa*, forming a sister subclade to the subclade of *D. strigosa* MF179495 (from Yunan, China; Yu et al., 2017) and *D. dumetorum* (Figure 7). Notably, both trees strongly supported (BS = 100% and PP = 1) two super‐clades, which contained clade III–IV and clade VII–XI–IX and four clades, including clade III, VII, XI, and IX (Figure 7). Moreover, phylogenetic statuses of some *Diospyros* species endemic to China were recognized, for instance, *D. maclurei* was belonged to clade VII, and *D. hainanensis*, *D. xishuangbannaensis* and *D. sutchuensis* could been found in clade XI.

### 3.5 Selective pressure in CDS genes

**FIGURE 8:**
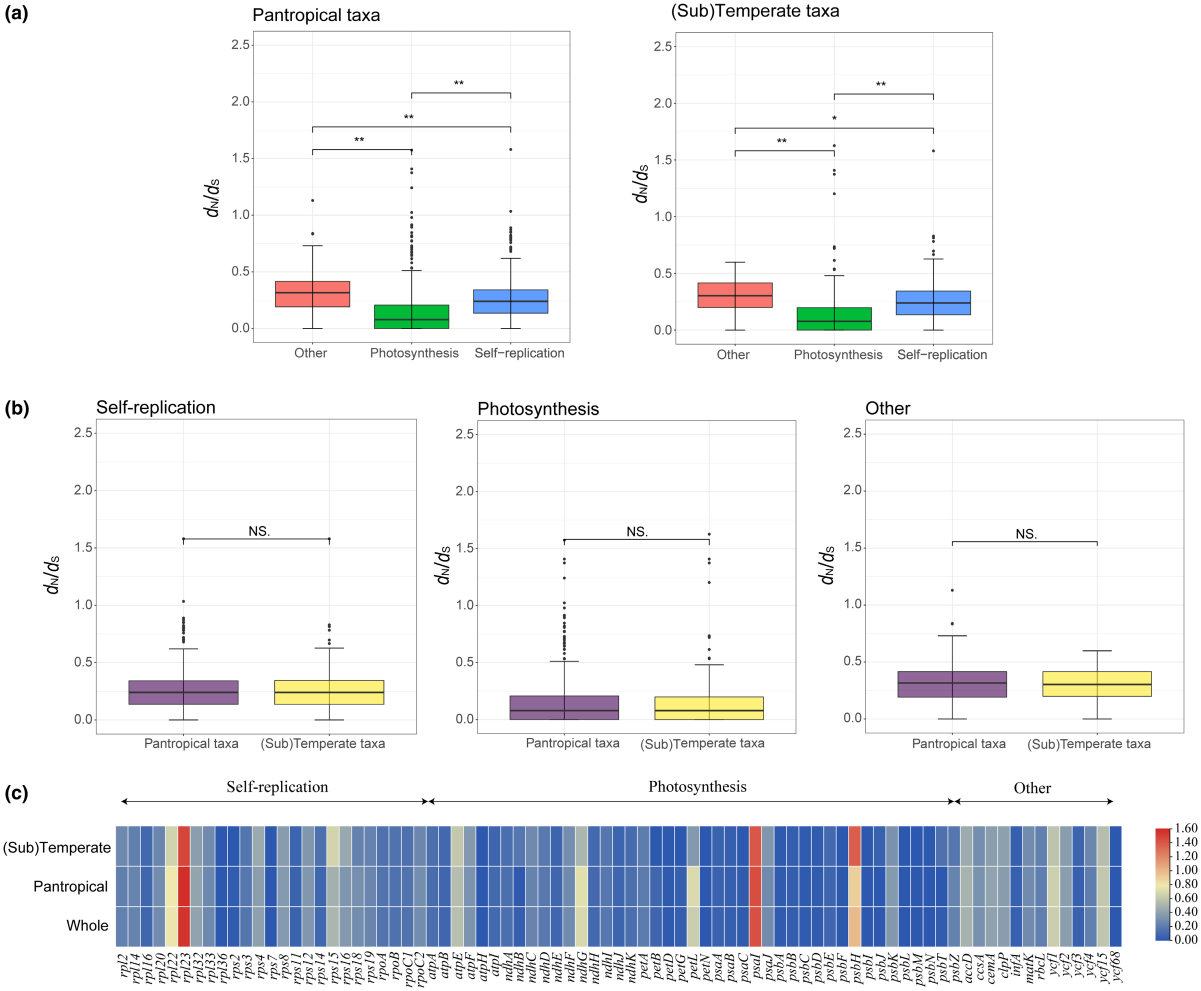
Analyses of evolutionary pressure on plastid gene homologs in 45 *Diospyros* species. (a) A comparison of *d*_N_/*d*_S_ values among photosynthesis‐correlated genes, self‐replication‐correlated genes, and other protein coding genes in the (sub)temperate and pantropical species. (b) A comparison of *d*_N_/*d*_S_ values among gene homologs from the (sub)temperate and pantropical species for photosynthesis‐correlated genes, self‐replication‐correlated genes, and other protein coding genes. *p < .05; **p < .01. (c) A heatmap showing the *d*_N_/*d*_S_ values of CDS genes within the (sub)temperate and pantropical species.

Then, to evaluate the evolutionary forces acting on the protein‐coding homologous genes in the 45 *Diospyros* species, the *d*_N_/*d*_S_ values of 81 CDS genes were calculated (Table S6a). Our results showed the *d*_N_/*d*_S_ values of 78 CDS genes were less than 1, indicating that most homologous genes were under purifying selection. However, we also found that the self‐replication gene *rpl*23 and the photosynthesis gene *psa*I (*d*_N_/*d*_S_ > 1) were under strong positive selection in both pantropical and (sub)temperate taxa. Remarkably, the *d*_N_/*d*_S_ value of the photosynthesis gene *psb*H was higher than one only in the (sub)temperate taxa (Figure 8c, Table S6a). For species in the pantropical and (sub)temperate taxa, the *d*_N_/*d*_S_ values of photosynthesis‐related genes were significantly lower than self‐replication‐related and the other genes, suggesting stronger purifying selection (Figure 8a, Table S6b). However, there were no significant difference of *d*_N_/*d*_S_ values in self‐replication‐related, photosynthesis‐related and the other genes between pantropical and (sub)temperate taxa, respectively (Figure 8b, Table S6c).

### 3.6 Codon usage bias

The comparison of the occurrence frequencies of different codons in the 45 *Diospyros* plastomes identified leucine (Leu) as the most used amino acid (10.32%), and its encoding codon UUA with a maximum RSCU value of 1.95 accounted for 3.36% of all the codons (Table S7). Codon GCU (RSCU = 1.83) and AGA (RSCU = 1.82) were recognized as the second and third optimal codons, respectively (Table S7). On the contrary, cysteine (Cys) was the least used amino acid (1.06%), but serine (Ser) encoding codon AGC had a minimum RSCU value of 0.32 (Table S7). In addition, AUG and UGG encoding methionine (Met) and tryptophan (Trp) had an RSCU value of 1, indicating no bias in the codon usage for these two amino acids (Table S7). Moreover, 30 codons had an RSCU >1, of which 16 had U in its third position, 13 had A, and one had G, which indicates that the codons ending with U or A are preferred in the *Diospyros* plastomes (Table S7).

**FIGURE 9:**
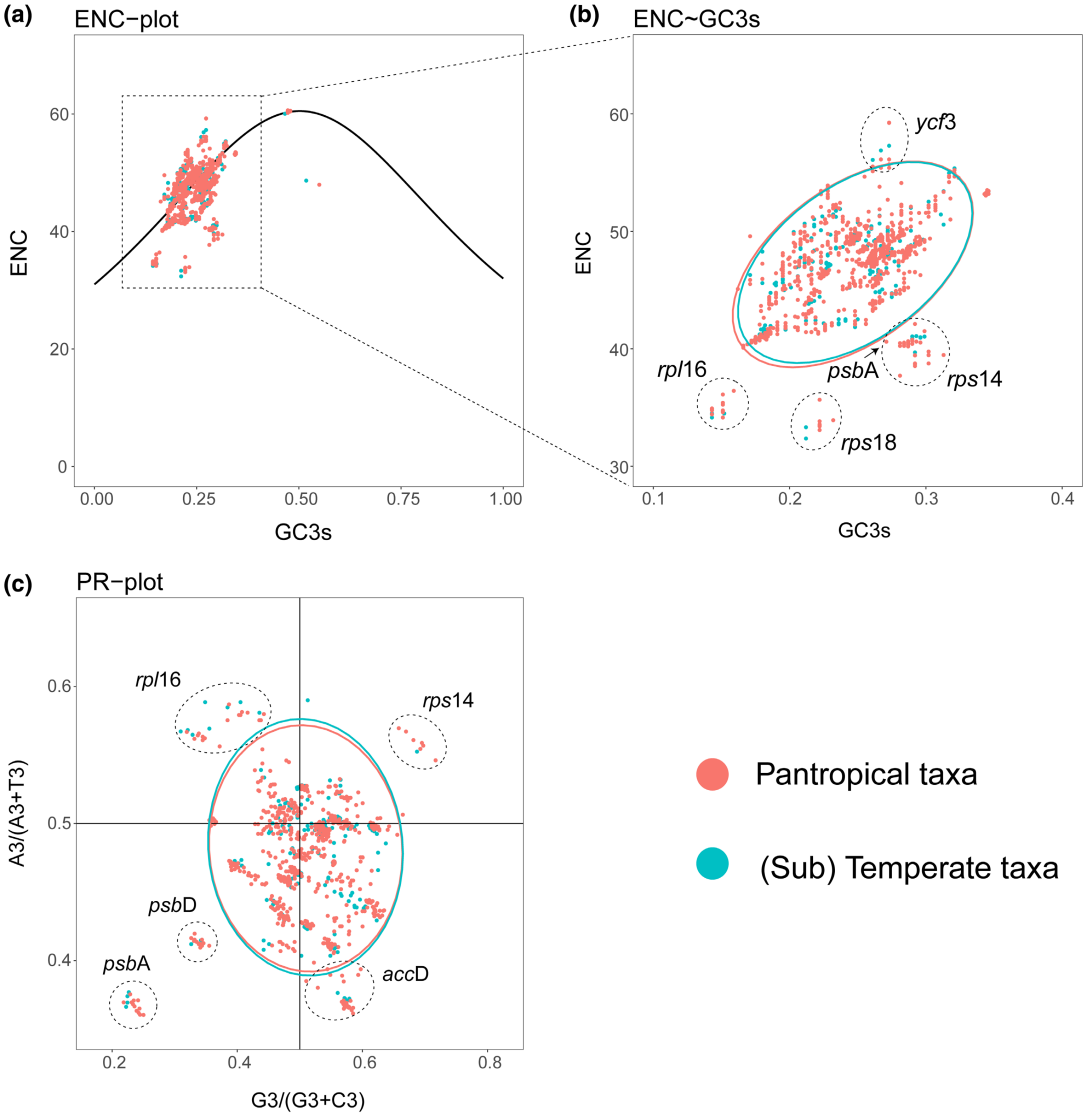
Effective number of codons (ENC) and PR2 plots of protein‐coding genes in plastomes of 45 *Diospyros* species. (a) ENC plots showing observed and expected and ENC values versus GC3s values of protein‐coding genes in the 45 *Diospyros* plastomes. (b) Comparison of ENC differences in two different climate zones. (c) PR2 plots showing the base composition characteristics of protein‐coding genes in the 45 *Diospyros* plastomes. Red, genes of species from the (sub)temperate species; green, genes of species from the pantropical species.

Further, the ENC‐GC3 plot was obtained by taking the ENC value of each gene as the ordinate and the GC3 value as the abscissa to explore the kind of suffered stress (mutation pressure or natural selection; Figure 9). The ENC value ranged from 32.36 to 60.62 and the GC3 value from 0.143 to 0.346 (except *ycf* 68: 0.466–0.550; Table S8). Figure 9a shows that most genes are close to the standard curve, and a few are far below it, indicating the influence of mutation pressure and natural selection on the codon usage bias of *Diospyros* genes (Figure 9). Then, to accurately evaluate the difference between the observed value (ENC_obs_) and the expected value (ENC_exp_) of ENC, the (ENC_exp_ − ENC_obs_)/ENC_exp_ ratio was calculated (Table S6). The ENC frequency ranging from −0.1 to 0.1 indicated a slight difference between ENC_exp_ and ENC_obs_ values of most genes. The difference values in the codon usage bias of *Diospyros* genes was related to the difference in GC3, indicating a significant influence of mutation pressure on codon usage bias.

Detailed analysis showed considerable deviation in the observed ENC values from the standard curve for five genes (*rps*18, *rps*14, *psb*A, *rpl*16 and *ycf*3) of all the species (Figure 9a,b). Among all the genes, *ycf*3 showed the highest ENC value, while *rps*18 and *rpl*16 had the lowest (Figure 9b; Table S8). PR2 plot showed slight disequilibrium in A/T and G/C usage in the third codon position of CDSs of the 45 *Diospyros* plastomes, especially these five CDSs (*acc*D, *psb*A, *psb*D, *rpl*16 and *rps*14; Figure 9c). More genes were distributed in Quadrant IV (at the right bottom of the Figure 9c) than the other three quadrants, indicating frequent use of G and T in the third codon position, particularly in gene *acc*D. This observation suggests that the existing codon usage pattern may be due to the combined action of natural selection and mutation. Genes from pantropical and (sub)temperate species were presented using different colors in the ENC and PR2 (parity rule 2) plots (Figure 9). There were no obvious potential differences in the main driving force of codon usage bias in *Diospyros* species between these two groups (Figure 9; Table S8).

## 4 DISCUSSION

### 4.1 Phylogenetic relationship of *Diospyros* species

So far, there is not a phylogenetic tree containing almost all *Diospyros* species (>500 spp.; Samuel et al., 2019) except the study of Duangjai et al. (2009), which provided the phylogenetic relationships of 119 *Diospyros* species based on eight plastid regions. Eleven clades were recognized but with relatively weak support rate in some clades (Duangjai et al., 2009). Recently, researchers have discussed using plastomes as super‐barcodes for phylogenetic studies (Li et al., 2021; Wang et al., 2017). The phylogenetic analysis of this study showed that the plastomes are helpful as a super‐barcode for the phylogeny relationship of *Diospyros* species (Figure 7). Two super‐clades which contained clade III–IV and clade VII–XI–IX were strongly supported (BS = 100% and PP = 1), which were 79% and 77% in the most‐parsimonious trees, respectively (Duangjai et al., 2009). The present phylogenetic analyses revealed that clade III, VII, XI, and IX were all totally monophyletic, with clade VII forming the basal clade of clade XI and IX, strongly supporting the previous phylogenetic analysis (Duangjai et al., 2009).

The present study found the topology of (sub)temperate *Diospyros* species was consistent with an earlier plastome‐based study (Li et al., 2018). However, we carried out the phylogenetic analysis using more samples and showed that revealed reliable results with greater precision. In the plastome‐based tree, *D. zhejiangensis* (2*n* = 2*x* = 30; Tang et al., 2019), *D. kaki* (2*n* = 6*x* = 90), the dioecious *D. deyangensis* (2*n* = 4*x* = 60; Zhang et al., 2016), and the polygamous *D. oleifera* shared a common furcation. Meanwhile, *D. glaucifolia* and *D. lotus* were genetically close to *D. morrisiana*, identical to the classification based on phenotypic characteristics (Lee et al., 1996), which is similar to Tang et al. (2014). In addition to the similar phylogenetic relationships among the three species, *D. morrisiana* has relatively smaller leaves and fruits than *D. glaucifolia* and *D. lotus* (Lee et al., 1996). Meanwhile, *D. virginiana* was identified as the basal taxa of the (sub)temperate clade. The fruits of *D. virginiana* are an important food for wildlife, native people, and Euro‐American colonists. These fruits have never been commercialized, despite the selection of superior clones over the years (Boufford, 2022). Therefore, *D. virginiana*, as the base group of deciduous group and its wild existence, can be used as a species for cultivation and breeding.

In the pantropical taxa, *D. eriantha* and *D. strigosa* are clustered together based on new plastomes sequences, consisting with their similarities in morphological characteristics. However, the newly sequenced *D. strigosa* did not form a cluster with *D. strigosa* MF179495. We think that the latter sequenced plastome of *D. strigosa* MF179495 (collected from Yunan, China; Yu et al., 2017) was most likely misidentified, considering that *D. strigosa* is found only in Hainan, China (Lee et al., 1996). In the clade VII and XI, we recognized phylogenetic positions of some potential *Diospyros* species endemic to China, for example, *D. maclurei* endemic to Hainan was belonged to clade VII; *D. hainanensis* was located at the base of clade XI, which is found only in Guandong and Hainan; *D. xishuangbannaensis* was likely close to *D. olen*; *D. sutchuensis* was genetically close to *D. rhombifolia* and *D. cathayensis*. Finally, *D. ferrea* and 19 *Diospyros* species from New Caledonia together made up a monophyletic clade III, which is consistent with the previous studies (Duangjai et al., 2009; Turner et al., 2016). Elucidating the boundaries between the different *Diospyros* species would improve our understanding of the cultivated species' origin, phylogeny and help decide the breeding strategy. However, except the (sub)temperate taxa, the pantropical taxa contains inadequate species in this study, and the phylogeny positions of some species are unresolved with week support (see Clade III&XI; Figure 7). In the future, the plastome‐based or nuclear genome‐based phylogenomic tree needs to be further studied based on more extensive sampling in *Diospyros*.

### 4.2 Adapted evolution of *Diospyros* plastomes

Furthermore, we found that the *d*_N_/*d*_S_ values of 78 common genes among the 45 *Diospyros* species were less than one. We also found that the *d*_N_/*d*_S_ values of photosynthesis‐related were significantly lower than self‐replication‐related and other genes in the (sub)temperate and pantropical taxa (Figure 8). This observation indicated that most important photosynthesis‐related genes are undergoing strong purifying selection. Purifying selection usually reduces genetic diversity and maintain gene homozygosity via the selective removal of deleterious alleles (Cvijović et al., 2018). Moreover, the functional importance of a protein determines its evolutionary rate (Wang et al., 2011). However, calculation of pairwise *d*_N_/*d*_S_ of the photosynthesis gene *psb*H still showed positive selection signal in (sub)temperate species. This result indicates that the *psb*H gene is likely to be involved in the adaptation to (sub)temperate zone. However, a small portion of total DNA represented by organelle genomes, such as plastomes, cannot fully display a large number of selected sites and results from 45 *Diospyros* species are inadequate for the entire genus. Therefore, a nuclear, genome‐wide transcriptome approach and more extensive sampling are necessary to confirm the selection pressure on *Diospyros* species for future research.

Typically, the usage pattern of the third base of the codon is closely related to codon usage bias (Gao et al., 2022). The GC composition is closely related with codon and amino acid usage, and the GC content of the third base of a codon (GC3) reflects codon usage patterns (Chen et al., 2013). Studies have shown that dicots and monocots prefer to use A/U and C/G as ending codons, respectively (Liu et al., 2020; Yao et al., 2008). Our study found that the average GC content and GC3 values of *Diospyros* codons were 37.7%–38.2% and 14.3%–34.6% (except *ycf* 68: 44.6%–55.0%), respectively, indicating that the major *Diospyros* codons also preferred A/T(U) in the third position, which is consistent with the RSCU values of *Diospyros* genes.

Mutation pressure and natural selection are the major factors influencing codon usage bias in any organism (Rao et al., 2011; Sharp et al., 2010). However, the main factors affecting codon usage bias vary significantly among species. According to the parity rule 2 analysis, the GT content at the third position of a codon is higher than AC content. However, A and T were used more frequently than G and C in the third position of the codons of *Diospyros* genes (Table S8), which suggested natural selection as one of the main reasons for *Diospyros* codon usage bias. Further ENC‐plot analysis showed that the ENC value of most genes was close to the expected value (Figure 8a), suggesting that the codon usage bias of these genes was related to GC3, and mutation was the main influencing factor. Additionally, a few genes in the plot (*rpl*16, *rps*18 and *rps*14) were well below the expected curve (Figure 8b), indicating the influence of natural selection on the codon deviations of these genes. Integrated analysis of the ENC‐plot and PR2 plot revealed that mutation and natural selection jointly affected the codon usage bias of *Diospyros* genes, and mutation pressure played a significant role, consistent with the reports on CDS genes in *Oncidium* (Xu et al., 2011) and the findings in Rosaceae (Liu et al., 2021). Moreover, studies in *Drynaria* also indicated mutation pressure as the driving force of codon usage bias (Shen et al., 2021). However, Sheng et al. (2021) reported natural selection as the main factor influencing codon usage bias of five different *Miscanthus* species. These results suggest that various pressures influence plastomes, and codon usage preferences of plastome genes vary among the dicotyledon taxa.

### 4.3 Potential ptDNA barcodes and phylogeny of *Diospyros*

Taxonomic classification is challenging in *Diospyros* (Lee et al., 1996). Moreover, the worldwide distribution and phenotypic plasticity make it difficult to identify the wild *Diospyros* species (Ebenaceae; Lin et al., 2020). Generally, in such cases barcodes are used. However, only a limited number of DNA barcodes (e.g., *rbc*L, *mat*K, and *trn*H‐*psb*A) are available to resolve the phylogenetic relationships among the groups (Duangjai et al., 2009; Linan et al., 2019). Therefore, comparing more plastomes for developing variable DNA barcodes is important for *Diospyros* species. Generally, the mutational hotspots have the potential to resolve taxonomic issues. They provide adequate genetic information for species identification and, therefore, can be used to develop novel DNA barcodes. The four potential mutational hotspots (ccs*A‐ndhD*, *trn*T‐*trn*L, *rps*16‐*psb*K, *pet*A‐*psb*J) identified in this study could be suitable barcodes for *Diospyros* classification. In addition, five other potential mutational hotspots (*rpl*33, *rpl*22, *petL*, *rps*15 and *ycf*1) were identified with high nucleotide polymorphisms in CDS. By comparison, in a previous study on *Diospyros*, eight potential mutational hotspots (*trn*H‐*psb*A, *rps*16‐*trn*Q, *rpo*B‐*trn*C, *rps*4‐*trn*T‐*trn*L, *ndh*F, *ndh*F‐*rpl*32‐*trn*L, *ycf*1a and *ycf*1b) showed high divergence in plastomes and were recommended as core DNA barcodes (Li et al., 2018). Of these, *ycf*1 has been widely applied in plant phylogeny and DNA barcoding studies (Dastpak et al., 2018; Parks et al., 2011; Yang et al., 2017). *Trn*H‐*psb*A, *trn*L‐*trn*F‐*ndh*J, *pet*A‐*psb*J and *rps*16‐*trn*Q have also been used for phylogenetic studies (Shaw et al., 2005, 2007). Meanwhile, ccs*A‐ndhD*, *rps*16‐*psb*K, *pet*A‐*psb*J, *rpl*33, *rpl*22, *petL*, *psaC* and *rps*15 are novel hotspots identified as potential barcodes in this study.

## 5 CONCLUSION

The present study analyzed the plastome sequences of 45 *Diospyros* species and performed phylogenetic analysis to provide valuable genetic information. The findings based on this analysis partially supported the previous classifications based on morphological features. In addition, the study offers new insights into the phylogenetic relationships between (sub)temperate and pantropical taxa. Comparative plastome analysis revealed conserved genome structures and low nucleotide polymorphism. This study also identified mutational hotspots as phylogenetically informative markers that will contribute to future studies on *Diospyros* systematics and species identification. Moreover, this study assessed the adaptive evolution of the two taxa in *Diospyros* for the first time using *d*_N_/*d*_S_ values, ENC‐plot, and PR2 plot. This integrated analysis revealed natural selection and mutation pressure as the driving forces of plastomes of 45 *Diospyros* species. However, because of insufficient sampling, the current study did not provide adequate genetic information for understanding phylogenetic relationship and adaptive evolution for the entire *Diospyros* genus. Thus, we should focus on a more extensive sampling in future research.

## AUTHOR CONTRIBUTIONS

**Yue Huang:** Formal analysis (equal); software (equal); supervision (equal); validation (equal). **Qing Ma:** Data curation (lead); formal analysis (equal); funding acquisition (equal); investigation (lead); methodology (equal); project administration (equal); software (equal); supervision (equal); validation (equal); visualization (equal); writing – original draft (lead); writing – review and editing (lead). **Jing Sun:** Data curation (lead); investigation (lead); methodology (lead); software (lead); writing – original draft (equal). **Li‐Na Zhou:** Conceptualization (equal); data curation (equal); formal analysis (equal). **Chan‐Juan Lai:** Data curation (equal); software (equal). **Pan Li:** Conceptualization (lead); writing – review and editing (lead). **Xin‐Jie Jin:** Conceptualization (lead); data curation (lead); formal analysis (lead); funding acquisition (supporting); investigation (equal); methodology (equal); project administration (lead); resources (lead); software (equal); supervision (equal); validation (lead); visualization (lead); writing – original draft (lead); writing – review and editing (lead). **Yong‐Hua Zhang:** Conceptualization (lead); data curation (lead); formal analysis (lead); funding acquisition (lead); investigation (lead); methodology (lead); project administration (lead); resources (lead); software (lead); supervision (lead); validation (lead); visualization (lead); writing – original draft (lead); writing – review and editing (lead).

## FUNDING INFORMATION

This study was funded by the National Science Foundation of China (31800309), and the Zhejiang Provincial Public Welfare Technology and Application Research Project (LGN21C020007).

## CONFLICT OF INTEREST STATEMENT

None declared.

## Supporting information


Supplementary material

## Data Availability

The *Diospyros* plastomes generated in this study are available in the NCBI GenBank repository with accession numbers OP480008, OP480009 and OP485441. The supplementary material can be download all files using the URL: https://datadryad.org/stash/share/JglK1pjqccr7Fylxl45HtlXMztzhrqoEaXFfbZktSCo.
