# Supplementary material for: Comparative analysis of Diospyros (Ebenaceae) plastomes: Insights into genomic features, mutational hotspots, and adaptive evolution
Source: Ecol Evol. 2023 Jul 12;13(7):e10301. doi: 10.1002/ece3.10301 (PMC10338900; doi:10.1002/ece3.10301)
Supplement: Supplementary file 1 — Supplementary material [file ECE3-13-e10301-s001.zip › Supplementary file/Figure S1.docx]

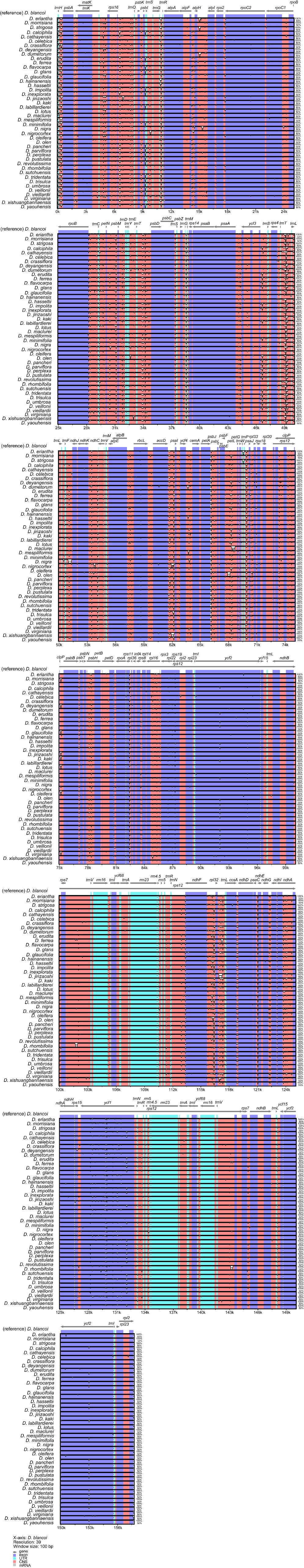


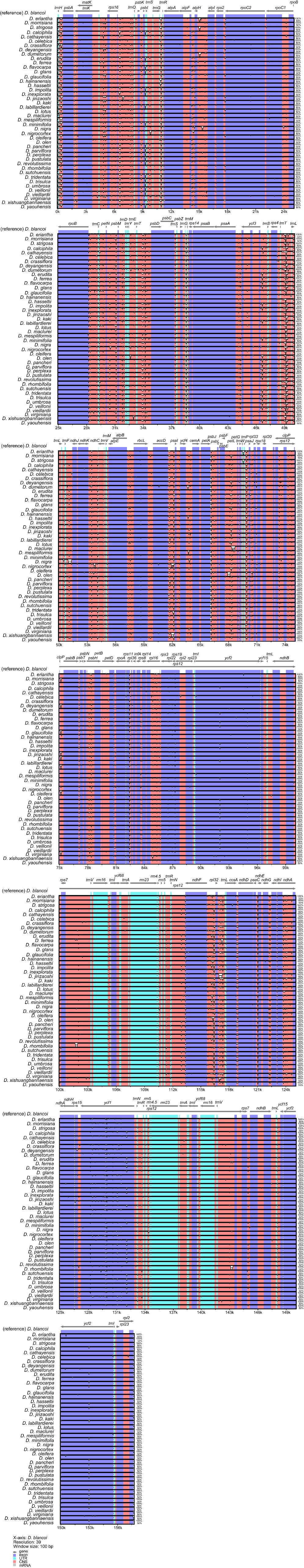


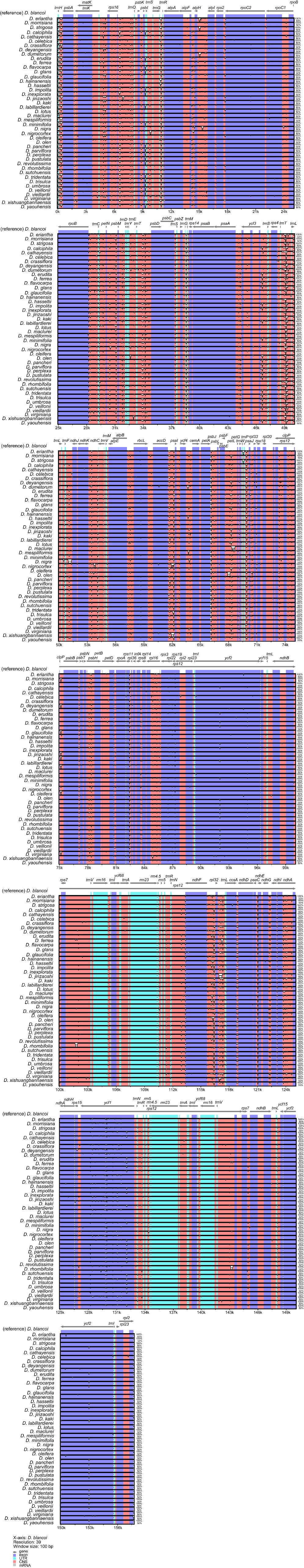


Fig. S1. Multiple alignment of 45 *Diospyros* plastomes using mVISTA. The grey arrows above the alignment indicate the gene names and transcription direction. The overall percentage of sequence similarity is indicated on the right with 50% to 100% consistency. Purple, exons; light blue, introns; Pink, non-coding sequences.
