# Supplementary material for: Comparative analysis of Diospyros (Ebenaceae) plastomes: Insights into genomic features, mutational hotspots, and adaptive evolution
Source: Ecol Evol. 2023 Jul 12;13(7):e10301. doi: 10.1002/ece3.10301 (PMC10338900; doi:10.1002/ece3.10301)
Supplement: Supplementary file 1 — Supplementary material [file ECE3-13-e10301-s001.zip › Supplementary file/Figure S1.pdf]

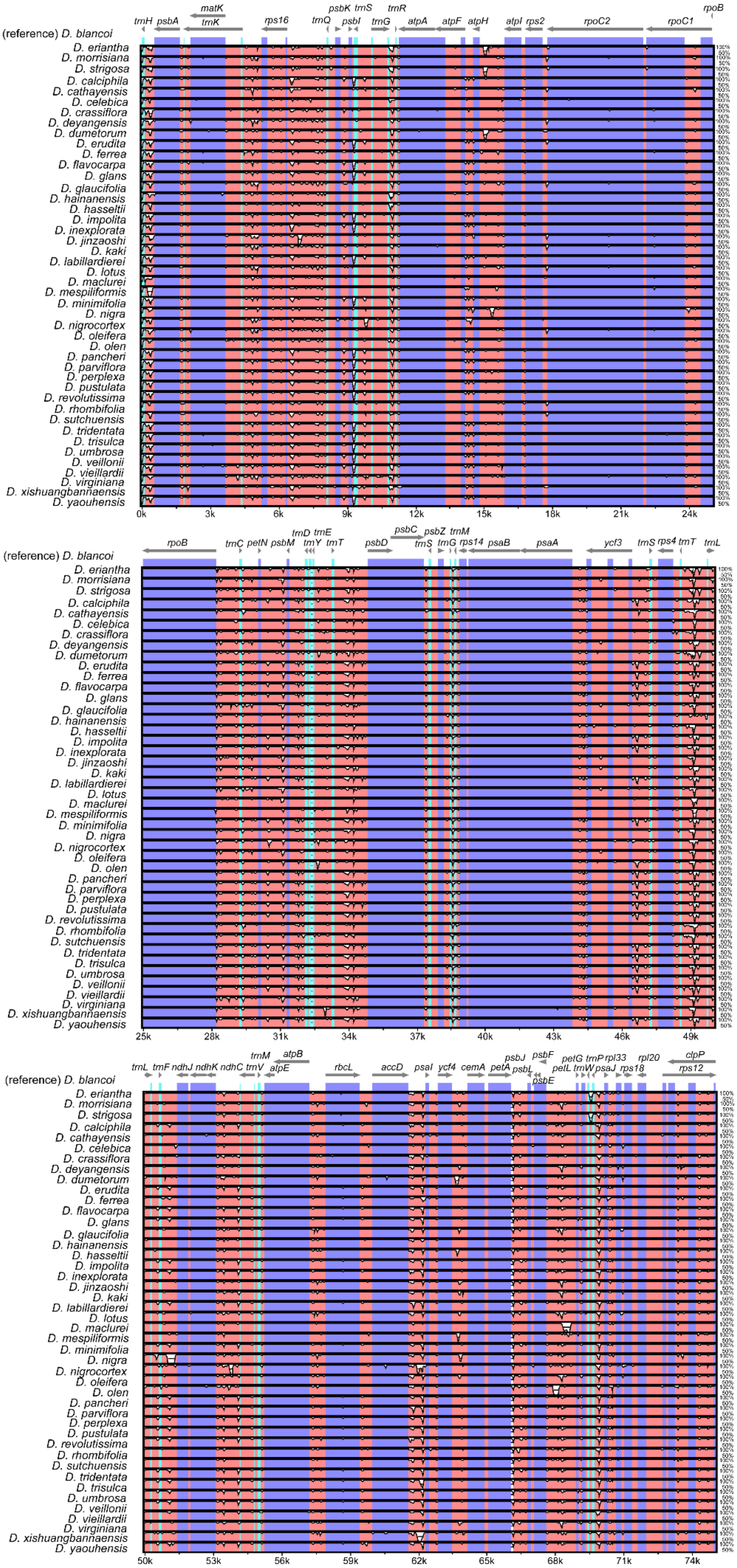
(reference) D. blancoi
D. eriantha
D. morrisiana
D. strigosa
D. calciphila
D. cathayensis
D. celebica
D. crassiflora
D. deyangensis
D. dumetorum
D. erudita
D. ferrea
D. flavocarpa
D. glans
D. glaucifolia
D. hainanensis
D. hasseltii
D. impolita
D. inexplorata
D. jinzaoshi
D. kaki
D. labillardierei
D. lotus
D. maclurei
D. mespiliformis
D. minimifolia
D. nigra
D. nigrocortex
D. oleifera
D. olen
D. pancheri
D. parviflora
D. perplexa
D. pustulata
D. revolutissima
D. rhombifolia
D. sutchuensis
D. tridentata
D. trisulca
D. umbrosa
D. veillonii
D. vieillardii
D. virginiana
D. xishuangbannaensis
D. yaouhensis
trnH psbA matK trnK rps16 trnQ psbK psbI trnS trnG trnR atpA atpF atpH atpI rps2 rpoC2 rpoC1 rpoB
0k 3k 6k 9k 12k 15k 18k 21k 24k
rpoB trnC petN psbM trnD trnY trnE trnT psbD psbC psbZ trnS trnG trnM rps14 psaB psaA ycf3 trnS rps4 trnT trnL
25k 28k 31k 34k 37k 40k 43k 46k 49k
trnL trnF ndhJ ndhK ndhC trnV trnM atpB atpE rbcL accD psaI ycf4 cemA petA psbJ psbL psbF psbE petG petL trnW trnP psaJ rpl33 rps18 rpl20 clpP rps12
50k 53k 56k 59k 62k 65k 68k 71k 74k
100%
50%

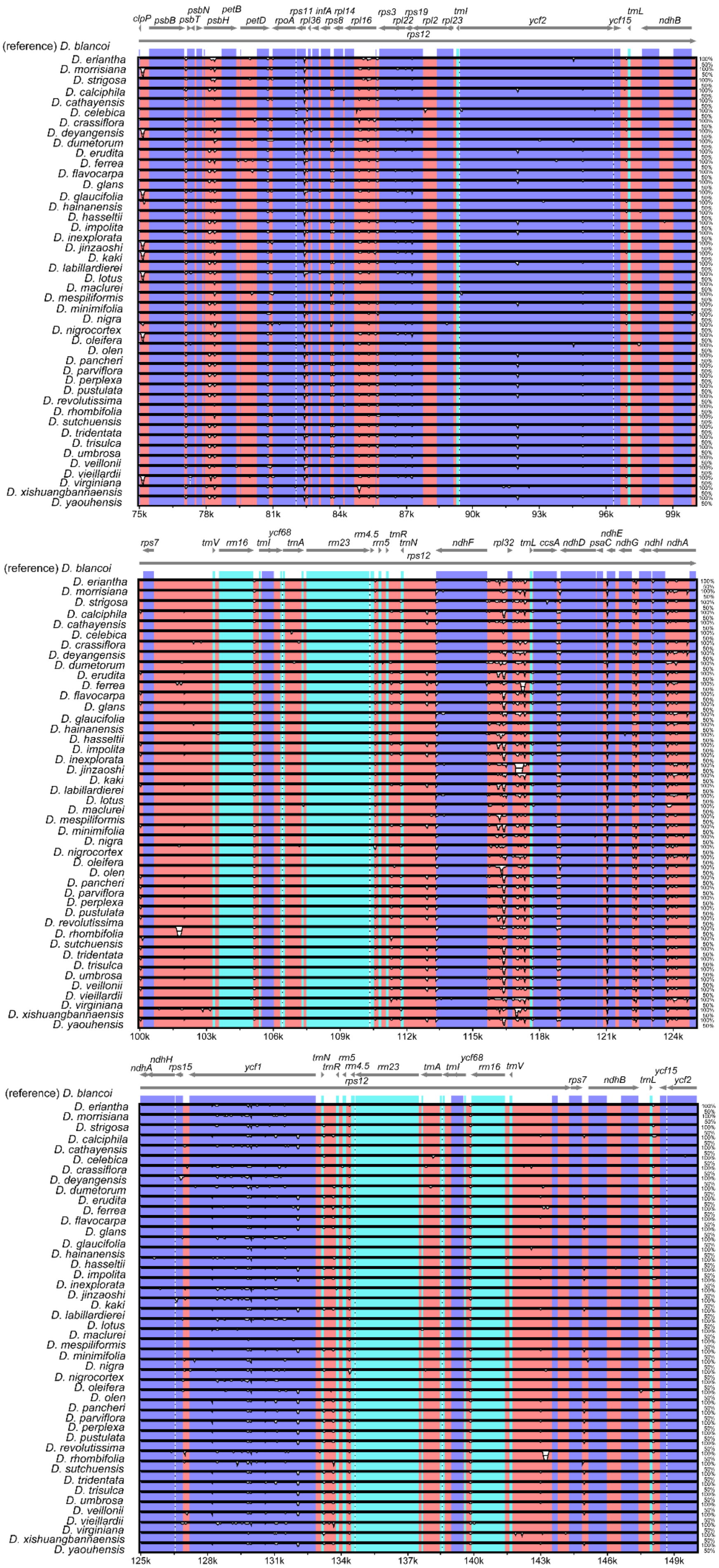

clpP psbB psbT psbN psbH petB petD rpoA rps11 rpl36 infA rps8 rpl14 rpl16 rps3 rpl22 rps19 rpl2 rpl23 trnI ycf2 ycf15 trnL ndhB
rps12
(reference) D. blancoi
D. eriantha
D. morrisiana
D. strigosa
D. calciphila
D. cathayensis
D. celebica
D. crassiflora
D. deyangensis
D. dumetorum
D. erudita
D. ferrea
D. flavocarpa
D. glans
D. glaucifolia
D. hainanensis
D. hasseltii
D. impolita
D. inexplorata
D. jinzaoshi
D. kaki
D. labillardierei
D. lotus
D. maclurei
D. mespiliformis
D. minimifolia
D. nigra
D. nigrocortex
D. oleifera
D. olen
D. pancheri
D. parviflora
D. perplexa
D. pustulata
D. revolutissima
D. rhombifolia
D. sutchuensis
D. tridentata
D. trisulca
D. umbrosa
D. veillonii
D. vieillardii
D. virginiana
D. xishuangbannaensis
D. yaouhensis
75k 78k 81k 84k 87k 90k 93k 96k 99k
rps7 trnV rrn16 ycf68 trnI trnA rrn23 rrn4.5 rrn5 trnR trnN rps12 ndhF rpl32 trnL ccsA ndhD ndhE psaC ndhG ndhI ndhA
100k 103k 106k 109k 112k 115k 118k 121k 124k
ndhA ndhH rps15 ycf1 trnN trnR rrn5 rrn4.5 rrn23 rps12 trnA trnI ycf68 rrn16 trnV rps7 ndhB trnL ycf15 ycf2
125k 128k 131k 134k 137k 140k 143k 146k 149k
100% 50%

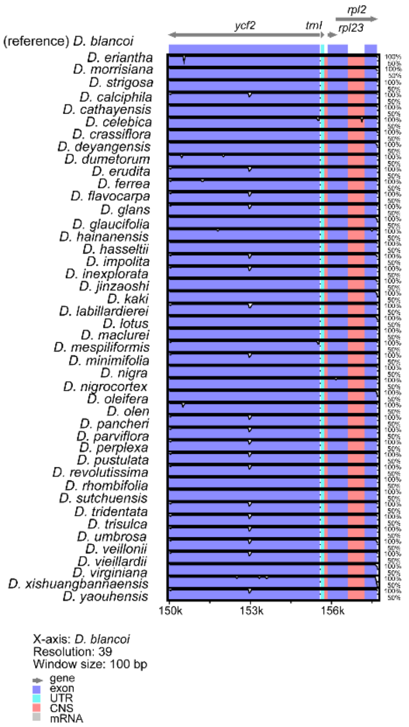

Fig. S1. Multiple alignment of 45 *Diospyros* plastomes using mVISTA. The grey arrows above the alignment indicate the gene names and transcription direction. The overall percentage of sequence similarity is indicated on the right with 50% to 100% consistency. Purple, exons; light blue, introns; Pink, non-coding sequences.
