# Supplementary material for: Comparative analysis of Diospyros (Ebenaceae) plastomes: Insights into genomic features, mutational hotspots, and adaptive evolution
Source: Ecol Evol. 2023 Jul 12;13(7):e10301. doi: 10.1002/ece3.10301 (PMC10338900; doi:10.1002/ece3.10301)
Supplement: Supplementary file 1 — Supplementary material [file ECE3-13-e10301-s001.zip › Supplementary file/Figure S2.docx]

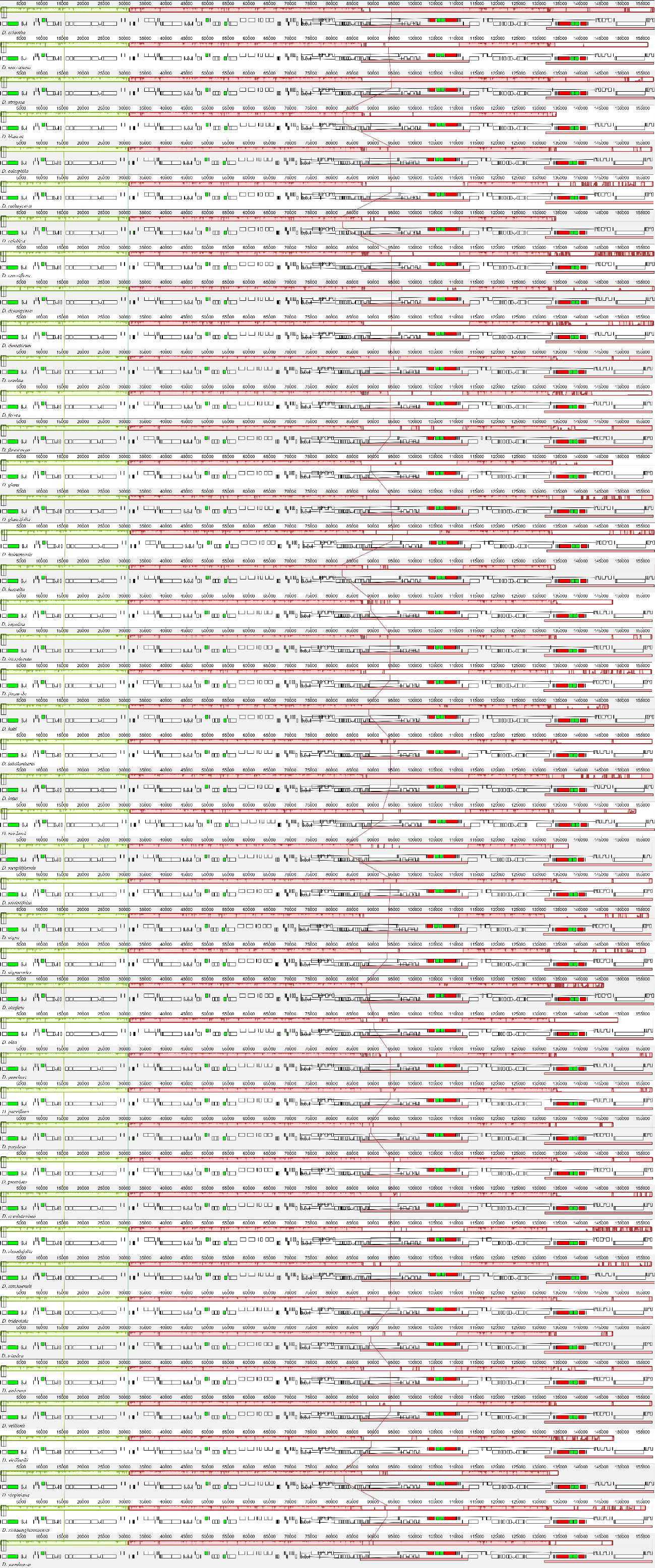


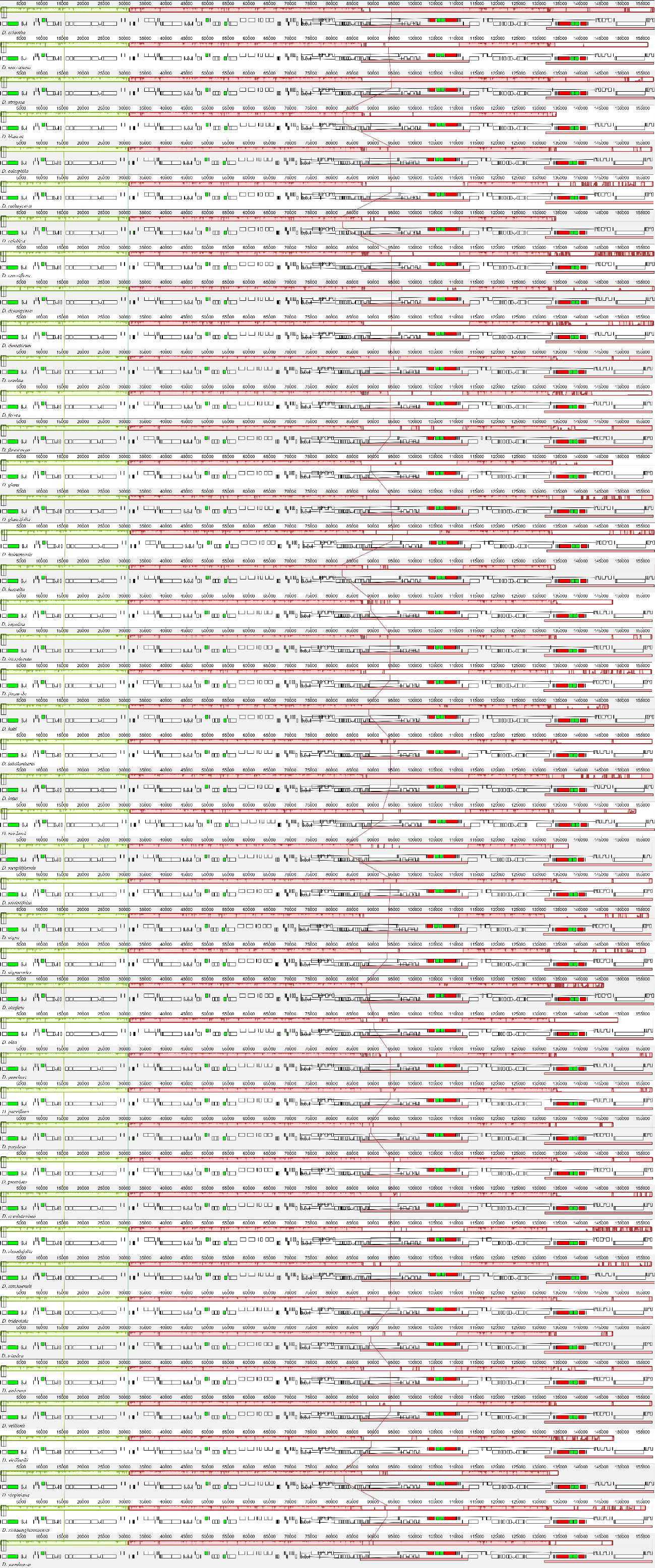


Fig. S2. Whole genome alignment of 45 *Diospyros* plastomes using Mauve algorithm. The overall sequence similarities were shown above and the collinear DNA components were indicated by the green, red, and white blocks below.
