# Supplementary material for: Comparative analysis of Diospyros (Ebenaceae) plastomes: Insights into genomic features, mutational hotspots, and adaptive evolution
Source: Ecol Evol. 2023 Jul 12;13(7):e10301. doi: 10.1002/ece3.10301 (PMC10338900; doi:10.1002/ece3.10301)
Supplement: Supplementary file 1 — Supplementary material [file ECE3-13-e10301-s001.zip › Supplementary file/Figure S2.pdf]

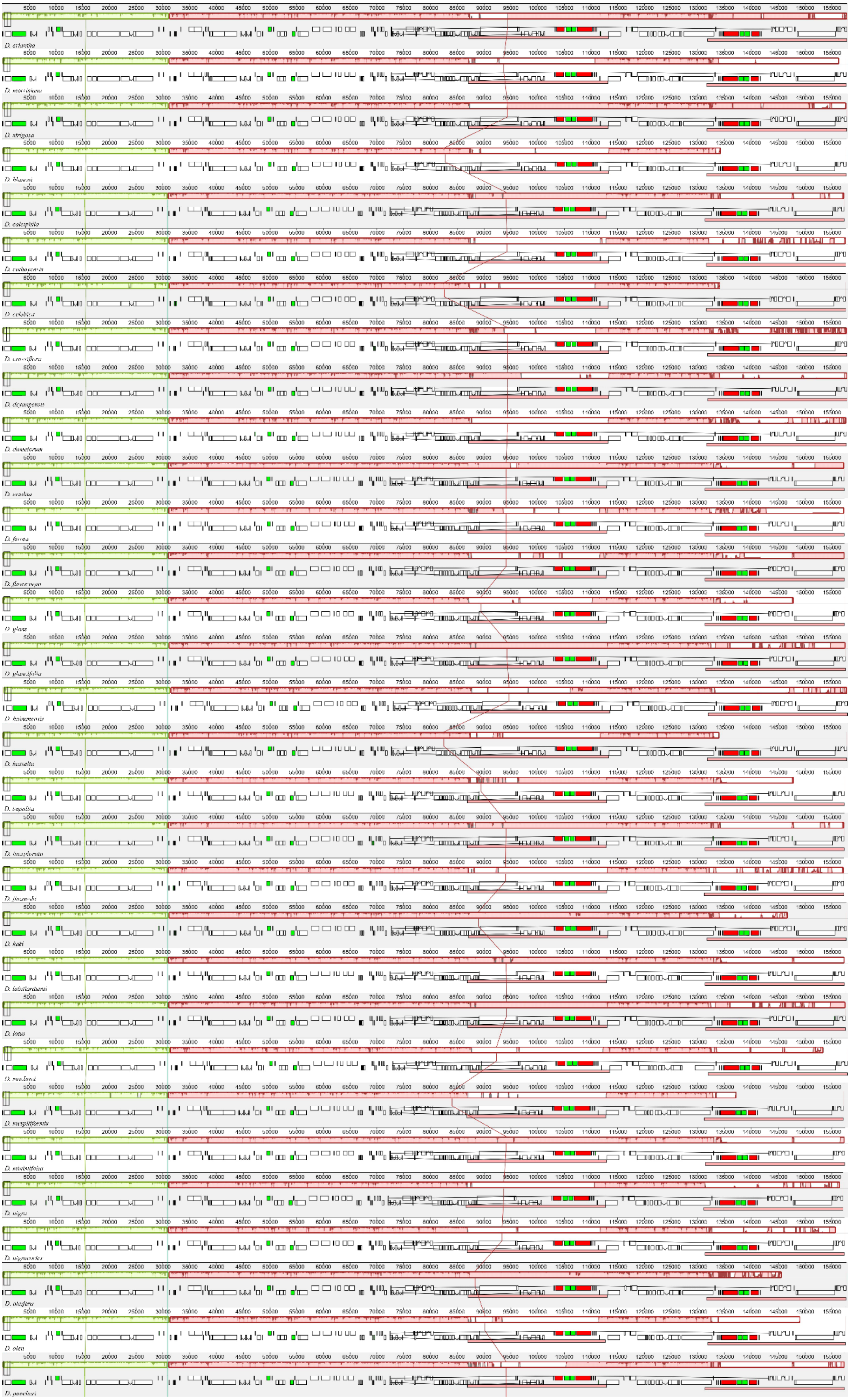
5000
10000
15000
20000
25000
30000
35000
40000
45000
50000
55000
60000
65000
70000
75000
80000
85000
90000
95000
100000
105000
110000
115000
120000
125000
130000
135000
140000
145000
150000
155000
D. eriantha
D. strigosa
D. celebica
D. crassiflora
D. dumetorum
D. ferrea
D. glaucifolia
D. hainanensis
D. hasseltii
D. kaki
D. labillardierei
D. lotus
D. mespiliformis
D. nigra
D. oleifera

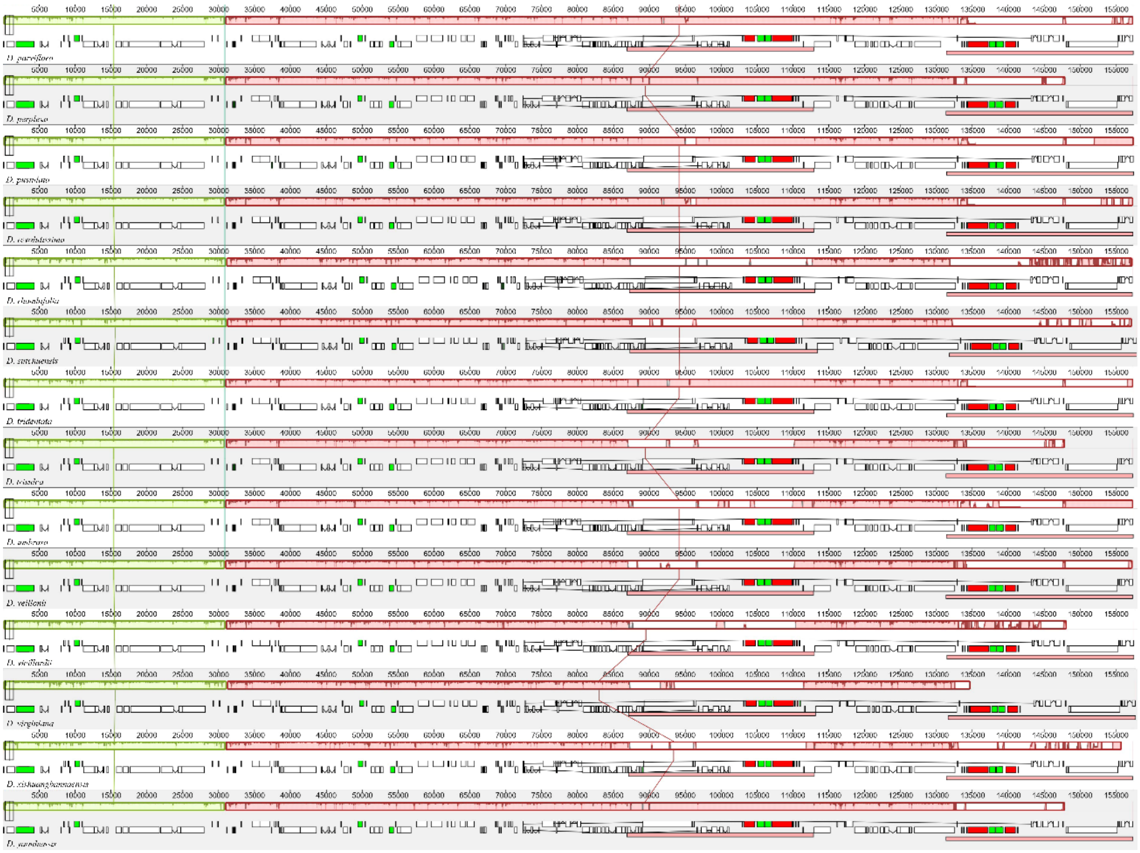

Fig. S2. Whole genome alignment of 45 *Diospyros* plastomes using Mauve algorithm. The overall sequence similarities were shown above and the collinear DNA components were indicated by the green, red, and white blocks below.
