# Supplementary material for: Comparative analysis of Diospyros (Ebenaceae) plastomes: Insights into genomic features, mutational hotspots, and adaptive evolution
Source: Ecol Evol. 2023 Jul 12;13(7):e10301. doi: 10.1002/ece3.10301 (PMC10338900; doi:10.1002/ece3.10301)
Supplement: Supplementary file 1 — Supplementary material [file ECE3-13-e10301-s001.zip › Supplementary file/Table S1.docx]

**Table** **S1** List of genes in the plastomes of *Diospyros.*

| Gene category | Groups | Name |
| --- | --- | --- |
| Self-replication | Transfer RNA | *trn*A-UGC^a*,b^(×2), *trn*C-GCA, *trn*D-GUC, *trn*E-UUC, *trn*F-GAA, *trn*G-UCC, *trn*G-UCC^a*^, *trn*H-GUG, *trn*I-CAU^b^(×2), *trn*I-GAU^a*,b^(×2), *trn*K-UUU^a*^, *trn*L-CAA^b^(×2), *trn*L-UAA^a*^, *trn*L-UAG, *trn*M-CAU(×2), *trn*N-GUU^b^(×2), *trn*P-UGG, *trn*Q-UUG, *trn*R-ACG^b^(×2), *trn*R-UCU, *trn*S-GCU, *trn*S-GGA, *trn*S-UGA, *trn*T-GGU, *trn*T-UGU, *trn*V-GAC^b^(×2), *trn*V-UAC^a*^, *trn*W-CCA, *trn*Y-GUA |
|  | Ribosome RNA | *rrn*4.5^b^(×2), *rrn*5^b^(×2), *rrn*16^b^(×2), *rrn*23^b^(×2) |
|  | Large subunit | *rpl*2^a*,b^(×2) , *rpl*14, *rpl*16^a*^, *rpl*20, *rpl*22, *rpl*23^b^(×2), *rpl*32, *rpl*33, *rpl*36 |
|  | Small subunit | *rps*2, *rps*3, *rps*4, *rps*7^b^(×2), *rps*8, *rps*11, *rps*12^a**,b^(×2), *rps*14, *rps*15, *rps*16^a*^, *rps*18, *rps*19, Ψ*rps*19^b^ (a short one in most *Diospyros*) |
|  | RNA polymerase | *rpo*A, *rpo*B, *rpo*C1^a*^, *rpo*C2 |
| Photosynthesis | ATP synthase | *atp*A, *atp*B, *atp*E, *atp*F^a*^, *atp*H, *atp*I |
|  | NADH dehydrogenase | *ndh*A^a*^, *ndh*B^a*,b^(×2), *ndh*C, *ndh*D, *ndh*E, *ndh*F, *ndh*G, *ndh*H, *ndh*I, *ndh*J, *ndh*K |
|  | Cytochrome | *pet*A, *pet*B^a*^, *pet*D^a*^, *pet*G, *pet*L, *pet*N |
|  | Photosystem I | *psa*A, *psa*B, *psa*C, *psa*I, *psa*J |
|  | Photosystem II | *psb*A, *psb*B, *psb*C, *psb*D, *psb*E, *psb*F, *psb*H, *psb*I, *psb*J, *psb*K, *psb*L, *psb*M, *psb*N, *psb*T, *psb*Z |
| Other genes | Rubisco | *rbc*L |
|  | Acetyl-CoA carboxylase | *acc*D |
|  | C-Type cytochrome synthesis | *ccs*A |
|  | Envelop membrane protein | *cem*A |
|  | Maturase | *mat*K |
|  | Protease | *clp*P^a**^ |
|  | Hypothetical reading frames | *ycf*1^b^, Ψ*ycf*1^b^ (a short one in all *Diospyros*), *ycf*2^b^(×2), *ycf*3 ^a**^, *ycf*4, *ycf*15^b^ (×2), *ycf*68^b^(×2) |
|  | Translation initiation factor | *inf*A |

a, genes containing introns; superscript *, the number of introns in the gene; b, genes located in IR regions, ×2, genes with two copies; Ψ, pseudogene.
