# Supplementary material for: Comparative analysis of Diospyros (Ebenaceae) plastomes: Insights into genomic features, mutational hotspots, and adaptive evolution
Source: Ecol Evol. 2023 Jul 12;13(7):e10301. doi: 10.1002/ece3.10301 (PMC10338900; doi:10.1002/ece3.10301)
Supplement: Supplementary file 1 — Supplementary material [file ECE3-13-e10301-s001.zip › Supplementary file/Table S2.docx]

**Table S2** Comparison of GC content of each part

| **Species** | **GC content of whole genome** | **GC content of LSC** | **GC content of SSC** | **GC content of IRs** | **GC content of CDS** |
| --- | --- | --- | --- | --- | --- |
| ***D. eriantha*** | 37.4 | 35.4 | 30.8 | 43.1 | 37.9 |
| ***D. morrisiana*** | 37.4 | 35.4 | 30.8 | 43.1 | 37.8 |
| ***D. strigosa*** | 37.4 | 35.4 | 30.8 | 43.1 | 37.9 |
| *D. blancoi* | 37.4 | 35.3 | 30.9 | 43.1 | 37.9 |
| *D. calciphila* | 37.4 | 35.4 | 30.7 | 43.1 | 37.7 |
| *D. cathayensis* | 37.4 | 35.4 | 30.8 | 43.0 | 37.9 |
| *D. celebica* | 37.4 | 35.4 | 30.8 | 43.1 | 37.9 |
| *D. crassiflora* | 37.4 | 35.4 | 30.8 | 43.1 | 37.9 |
| *D. deyangensis* | 37.4 | 35.4 | 30.8 | 43.1 | 37.9 |
| *D. dumetorum* | 37.3 | 35.3 | 30.8 | 43.0 | 37.8 |
| *D. erudita* | 37.4 | 35.4 | 30.7 | 43.1 | 37.7 |
| *D. ferrea* | 37.3 | 35.3 | 30.7 | 43.0 | 37.8 |
| 1. *flavocarpa* | 37.4 | 35.4 | 30.7 | 43.1 | 37.7 |
| *D. glans* | 37.4 | 35.4 | 30.7 | 43.1 | 37.7 |
| *D. glaucifolia* | 37.4 | 35.4 | 30.8 | 43.1 | 37.9 |
| *D. hainanensis* | 37.4 | 35.4 | 30.8 | 43.1 | 37.9 |
| *D. hasseltii* | 37.4 | 35.5 | 30.8 | 43.1 | 37.9 |
| *D. impolita* | 37.4 | 35.4 | 30.7 | 43.1 | 37.7 |
| *D. inexplorata* | 37.4 | 35.4 | 30.7 | 43.1 | 37.7 |
| *D. jinzaoshi* | 37.4 | 35.4 | 30.8 | 43.1 | 37.9 |
| *D. kaki* | 37.4 | 35.4 | 30.8 | 43.1 | 37.9 |
| *D. labillardierei* | 37.4 | 35.3 | 30.7 | 43.1 | 37.7 |
| *D. lotus* | 37.4 | 35.4 | 30.7 | 43.1 | 37.9 |
| *D. maclurei* | 37.4 | 35.4 | 30.8 | 43.1 | 38.2 |
| *D. mespiliformis* | 37.4 | 35.4 | 30.8 | 43.1 | 37.8 |
| *D. minimifolia* | 37.4 | 35.4 | 30.7 | 43.1 | 37.7 |
| *D. nigra* | 37.4 | 35.4 | 30.7 | 43.1 | 37.8 |
| *D. nigrocortex* | 37.4 | 35.3 | 30.7 | 43.1 | 37.8 |
| *D. oleifera* | 37.4 | 35.4 | 30.8 | 43.1 | 37.9 |
| *D. olen* | 37.4 | 35.5 | 31.2 | 43.1 | 37.9 |
| *D. pancheri* | 37.4 | 35.4 | 30.7 | 43.0 | 37.7 |
| *D. parviflora* | 37.4 | 35.4 | 30.7 | 43.1 | 37.8 |
| *D. perplexa* | 37.4 | 35.4 | 30.7 | 43.1 | 37.7 |
| *D. pustulata* | 37.4 | 35.4 | 30.7 | 43.1 | 37.7 |
| *D. revolutissima* | 37.4 | 35.4 | 30.7 | 43.1 | 37.7 |
| *D. rhombifolia* | 37.4 | 35.4 | 30.8 | 43.1 | 37.9 |
| *D. sutchuensis* | 37.4 | 35.4 | 30.9 | 43.0 | 37.9 |
| *D. tridentata* | 37.4 | 35.4 | 30.7 | 43.1 | 37.7 |
| *D. trisulca* | 37.4 | 35.4 | 30.7 | 43.1 | 37.7 |
| *D. umbrosa* | 37.4 | 35.4 | 30.7 | 43.1 | 37.7 |
| *D. veillonii* | 37.4 | 35.4 | 30.7 | 43.1 | 37.7 |
| *D. vieillardii* | 37.3 | 35.3 | 30.7 | 43.0 | 37.8 |
| *D. virginiana* | 37.4 | 35.4 | 30.9 | 43.0 | 37.9 |
| *D. xishuangbannaensis* | 37.4 | 35.5 | 30.7 | 43.1 | 37.9 |
| *D. yaouhensis* | 37.4 | 35.4 | 30.7 | 43.1 | 37.7 |
